# p38α (MAPK14) critically regulates the immunological response and the production of specific cytokines and chemokines in astrocytes

**DOI:** 10.1038/srep07405

**Published:** 2014-12-12

**Authors:** U. Lo, V. Selvaraj, J. M. Plane, O. V. Chechneva, K. Otsu, W. Deng

**Affiliations:** 1 Department of Biochemistry and Molecular Medicine, University of California, Davis, CA, USA; 2 Department of Cardiovascular Medicine, Osaka University Graduate School of Medicine, 2-2 Yamadaoka, Suita, Osaka, Japan; 3 Medical College, Hubei University of Arts and Science, Xiangyang, Hubei, China; 4 Shriners Institute for Pediatric Regenerative Medicine, Sacramento, CA, USA

## Abstract

In CNS lesions, “reactive astrocytes” form a prominent cellular response. However, the nature of this astrocyte immune activity is not well understood. In order to study astrocytic immune responses to inflammation and injury, we generated mice with conditional deletion of p38α (MAPK14) in GFAP+ astrocytes. We studied the role of p38α signaling in astrocyte immune activation both in vitro and in vivo, and simultaneously examined the effects of astrocyte activation in CNS inflammation. Our results showed that specific subsets of cytokines (TNFα, IL-6) and chemokines (CCL2, CCL4, CXCL1, CXCL2, CXCL10) are critically regulated by p38α signaling in astrocytes. In an in vivo CNS inflammation model of intracerebral injection of LPS, we observed markedly attenuated astrogliosis in conditional GFAPcre p38α^−/−^ mice. However, GFAPcre p38α^−/−^ mice showed marked upregulation of CCL2, CCL3, CCL4, CXCL2, CXCL10, TNFα, and IL-1β compared to p38αfl/fl cohorts, suggesting that in vivo responses to LPS after GFAPcre p38α deletion are complex and involve interactions between multiple cell types. This finding was supported by a prominent increase in macrophage/microglia and neutrophil recruitment in GFAPcre p38α^−/−^ mice compared to p38αfl/fl controls. Together, these studies provide important insights into the critical role of p38α signaling in astrocyte immune activation.

Astrocytes are the most abundant glial cell type in the CNS. They perform essential regulatory functions contributing to the maintenance of CNS homeostasis, including extracellular glutamate uptake^1^, K^+^ and H^+^ buffering^2^, energy metabolism^3^, synaptic transmission^4^, and microcirculation regulation^5^,^6^. The CNS is a relatively immune privileged compartment due to the lack of lymphatic drainage and unique characteristics of its vasculature. The blood brain barrier (BBB) regulates CNS microcirculation and consists of perivascular astrocytic endfeet constituting the neurovascular unit together with endothelial cells, pericytes and microglia. Disruption of the integrity of the BBB is one of the prominent pathological changes seen after CNS injury, and is induced by complex molecular mechanisms regulated by cytokines, chemokines, nitric oxide, adhesion molecules and matrix metalloproteinases (MMPs)^7^,^8^ that interact with components of the neurovascular unit. In particular, astrocytes in response to CNS injury exhibit a pathological hallmark termed as “reactive astrogliosis”. Studies of reactive astrocyte elimination suggest that astroglial scar formation ameliorates widespread tissue damage^9^,^10^, but detrimental effects by reactive astrocytes in inhibiting oligodendrocyte progenitor cell (OPC) migration and remyelination have also been reported^11^,^12^. Therefore, the outcome of reactive astrogliosis contributing to CNS neuropathology remains controversial. In response to inflammatory stimuli, astrocytes also produce proinflammatory cytokines and chemokines^13^,^14^,^15^,^16^. Studies suggest that astrocyte-derived TNFα and IL-1β exacerbate inflammatory responses by inducing BBB disruption through paracrine effects on endothelial cells^17^,^18^,^19^. Moreover, astrocytes are also implicated to be a major source of crucial chemokines involved in BBB disruption and leukocyte recruitment during CNS injury^14^,^20^. In particular, monocyte chemotaxis induced by CCL2 results in BBB breakdown by the downregulation of endothelial tight junction proteins^21^. CXCL10 promotes T lymphocyte infiltration into the CNS parenchyma^22^, whereas CXCL1 facilitates the transmigration of neutrophils across the BBB^23^. Taken together, studies on reactive astrogliosis and its regulation of immune cell trafficking suggest a significant yet controversial role of astrocytes during CNS inflammatory injury. It remains much debated as to whether immune activation of astrocytes can facilitate or exacerbate the outcome of CNS injury, and the definitive role of astrocytes contributing to CNS neuropathology remains to be determined.

The mitogen-activated protein kinase (MAPK) family transduces signals from the cell membrane to the nucleus in response to a wide variety of stimuli^24^. MAPK family members are serine/threonine protein kinases belonging to (1) p38 MAPKs, (2) extracellular signal-related kinases 1 and 2 (ERK1/2), and (3) Jun amino-terminal kinases (JNKs)^25^,^26^,^27^. p38 MAPK family members (p38α, p38β, p38γ and p38δ) are involved in cell cycle regulation, apoptosis, cell development, proliferation and inflammatory responses^28^,^29^,^30^,^31^,^32^. Among these, p38α is considered to be a central regulator of an inflammatory response in multiple cell types^33^,^34^. Downstream substrates of p38α include transcription factors and protein kinases, leading to divergent signaling cascades that dictate cellular responses to stress and inflammation^35^.

Pharmacological studies using p38 MAPK inhibitors indicated that astrocytes upregulate TNFα and IL-1β through a p38α-mediated pathway^36^,^37^. Through a feedback mechanism, downstream effects of TNFα and IL-1β promote subsequent upregulation of astrocyte-derived chemokines CCL2, CCL5 CXCL2, CXCL8 and CXCL10^15^,^38^,^39^. Although an essential role of p38α in the astrocyte immune responses has been suggested in *in vitro* studies using chemical inhibitors^37^,^40^,^41^, these inhibitors are not particularly specific and can also inhibit p38β and other enzymes. Therefore, the definitive role of p38α in the production of specific cytokines and chemokines in astrocytes has not been examined. Moreover, administration of p38 MAPK inhibitors *in vivo* also exhibits general therapeutic effects on CNS injury models^42^,^43^,^44^. However, due to the complexity of the *in vivo* CNS inflammation involving microglia, astrocytes and infiltrating leukocytes, there is insufficient information with regard to the cell-type specific immune response downstream of p38α signaling. Although astrocytes are capable of performing immunological functions during CNS injury, no studies have delineated the specific role of astrocytic p38α signaling in conditions of *in vivo* CNS inflammation. To study the p38α-mediated mechanisms in response to *in vivo* CNS inflammation, we first deleted the p38α gene in GFAP^+^ astrocytes in mice. Using this model, we sought to examine p38α-mediated immunological events during astrocytic immune response and define the role of astrocytes in CNS inflammation.

## Methods

### Animals

Mice with floxed alleles of p38α [B6.129-Mapk14<tm1.2Otsu>] were generated and described previously^45^. Human glial fibrillary acidic protein – cre recombinase expressing mice [hGFAP-Cre; FVB-Tg(GFAP-cre)25Mes/J] as well as the recombination-reporter strain ROSA26-tdTomato(mT)-EGFP(mG) [Gt(ROSA)26-Sortm4(ACTB-tdTomato,-EGFP)Luo/J] were obtained from the Jackson Laboratory (Bar Harbor, ME, USA). All mice were maintained in accordance to the National Institutes of Health's Guide for the Care and Use of Laboratory Animals. Experimental protocols used for this study were approved by the Institutional Animal Care and Use Committee at the University of California, Davis.

### Generation and genotyping of transgenic p38α^fl/fl^ and GFAP^cre^ p38α^−/−^ mice

Conditional GFAP-specific p38α knockout mice were generated using Cre/loxP recombination system by cross breeding hGFAP-Cre mice (FVB/N background) and p38α-floxed mice (C57BL/6 background). The hGFAP-Cre positive heterozygous p38α-floxed mice were then backcrossed to obtain mice homozygous for p38α (p38α^fl/fl^) mice with and without hGFAP-Cre (GFAP^cre^). Mice were genotyped using tail tip DNA at weaning. In brief, tail tips were lysed in buffer containing proteinase K and processed for DNA extraction by using QIAamp DNA Mini Kit (Qiagen, Valencia, CA). Purified genomic DNA was subjected to PCR using Biolase^®^ Taq DNA Polymerase (Bioline, Tauton, MA). The genotypes of p38α^fl/fl^ and GFAP^cre^p38α^−/−^ mice were examined by analyzing two transgenic compositions in genomic DNA, including hGFAP-Cre and the presence of p38α-floxed alleles using specific primers (Table S1). Desired genotypes of either p38α^fl/fl^ or GFAP^cre^p38α^−/−^ mice were generated for both *in vitro* primary culture and *in vivo* experiments.

### Primary culture of astrocytes from p38α^fl/fl^ and GFAP^cre^p38α^−/−^ mice

In order to obtain astrocyte cultures in high purity, we cross bred a double fluorescent Cre-reporter line, ROSA26-tdTomato(mT)-EGFP(mG)^46^, with hGFAP-Cre mice. After cross breeding, the tdTomato gene flanked by two loxP sites is deleted by Cre-loxP recombination, leading to EGFP expression in specific cell types with activated hGFAP promoter. We used the P2 progeny to generate astrocyte cultures. The primary astrocyte cultures were established from the neonatal cerebral cortex using a method modified from Fasano et al.^47^. In brief, the cortex was dissected and isolated from the diencephalon. Meninges and pia mater were carefully removed from the dorsal and ventral side of the cortex. Cerebral cortex was then separated and made into single cell suspension by gentle trituration followed by sequential passage through 100 μm and 40 μm nylon cell strainers. The resulting cells were then plated on 5% poly-D-lysine coated T75 flasks, and allowed to grow in minimum essential medium without D-valine (US Biologicals, Swampscott, MA) supplemented with 10% fetal bovine serum (FBS), 1% sodium pyruvate, 1% non-essential amino acid solution and 1% penicillin-streptomycin solution (all from Invitrogen, Carlsbad, CA). This mixed glial culture was maintained at 37°C in a humidified incubator with 5% CO_2_ for 7–10 days and allowed to reach 90% confluence. Subsequently, loosely adherent microglia and oligodendrocyte progenitor cells were dislodged and removed by mechanical shaking at 275 rpm for 20 hours in an orbital shaker within the incubator. The culture was then incubated in medium containing 50 mM L-leucine methyl ester hydrochloride for 1 hour to completely eliminate residual proliferating microglia^48^. The resulting cultures were then trypsinized, counted and passaged onto cover slips to examine the purity of astrocyte populations under fluorescent microscope. Astrocytes with activated hGFAP promoter will express EGFP-encoded green fluorescence protein on the membrane, whereas other contaminating cell types including microglia, oligodendrocytes, and fibroblasts will show a red fluorescent appearance due to an inactivated hGFAP promoter. The established protocol was then used to generate p38α^fl/fl^ and p38α^−/−^ astrocytes derived from p38α^fl/fl^ and GFAP^cre^p38α^−/−^ mice, respectively.

### In vitro astrocyte cytokine treatments

To examine the specific responses to interluekin-1β (IL-1β) and lipopolysaccharide (LPS) exposure, p38α^fl/fl^ and p38α^−/−^ astrocyte were seeded onto 35 mm 6-well plates at a density of 5 × 10^5^ cells per well. At > 90% confluence, astrocytes were treated with serum-free MEM medium for 24 hours and then exposed to IL-1β (100 ng/ml; Millipore, Billerica, MA) or LPS (100 ng/ml; Sigma, St. Louis, MO). For cytokine treatment studies, astrocytes were grouped into 3 wells per treatment and treated with either IL-1β for 0, 15, 30, 45, 60, 90 or 120 min, or LPS for 0, 30, 60 or 90 min to examine specific MAPK protein activation using western blot analysis.

To quantify different cytokine and chemokine gene expression, p38α^fl/fl^ and p38α^−/−^ astrocytes were seeded in 100 mm dishes at a density of 2.5 × 10^6^ cells per dish. Astrocytes were kept in culture for 2–4 days to reach ~90% confluence, and changed into serum-free MEM medium the day before cytokine treatment. To study the role of p38α signaling in astrocytic immune responses, we subjected astrocytes to IL-1β (30 ng/ml), interferon-γ (100 ng/ml, Millipore, Billerica, MA) or LPS (100 ng/ml) treatments for 0, 2, 4 or 6 hours, and examined immune gene expression using a real-time quantitative reverse transcriptase-polymerase chain reaction (qPCR) approach.

### Western blot analysis

Astrocytes treated with either IL-1β or LPS for indicative time points were grouped from 3 wells of 6-well plates and lysed by RIPA buffer supplemented with protease and phosphatase inhibitors (Roche, Indianapolis, IN). Cell lysates were resuspended by adding sample buffer containing 0.5 M Tris, sodium dodecyl sulphate (SDS), glycerol, dithiothreitol (DTT) and bromophenol blue. Samples were then boiled for 5 min, spun down to collect condensation and subjected to 10% SDS-PAGE. Gels were transferred to a PVDF membrane and immunoblotted with the following primary antibodies (from Cell Signaling Technology, Danvers, MA): phospho-p38 MAPK (Thr180/Tyr182) (Catalog # 4511), p38 MAPK (Catalog # 9212), phospho-SAPK/JNK (Thr183/Tyr185) (Catalog # 4668), SAPK/JNK (Catalog # 9295), phospho-p44/42 MAPK (Erk1/2)(Thr202/Tyr204) (Catalog # 9106), p44/42 MAPK (Erk1/2) (Catalog # 4695), MAPKAPK2 (MAP Kinase-activated protein kinase-2, Thr222) (Catalog # 3316), and α-tubulin (Santa Cruz Biotechnology, Santa Cruz, CA) (Catalog # sc-8035). In brief, PVDF membranes were blocked with 5% non-fat dry milk in Tris-buffered saline Tween 20 (TBST) for 1 hour at room temperature, and then incubated with primary antibodies in blocking solution for 16 hours at 4°C. After three washes in TBST, membranes were then incubated with horseradish peroxidase-conjugated secondary antibodies (GE Healthcare, Piscataway, NJ) for 1 hour at room temperature. Bound secondary antibodies were visualized by chemiluminescence using Pierce ECL Western Blotting Substrate (Thermo Scientific, Barrington, IL).

### Stereotaxic intracerebral LPS injection

Intracerebral injections of LPS into the cerebral cortex of p38α^fl/fl^ and GFAP^cre^ p38α^−/−^ mice were performed in a stereotaxic alignment system (David Kopf Instruments, Tujunga, CA). In brief, twelve-week old mice were anesthetized with isofluorane and positioned in the stereotaxic frame. A 0.2 mm hole was drilled in the skull of the right hemisphere at coordinates of 2 mm anterior to bregma and 1.5 mm lateral to the midline. Injection of LPS (2 μg in 2 μl saline) was performed using a glass micropipette at a depth of 1.8 mm below the duramater using the nanoject II (Drummond Scientific, Broomall, PA). After 6, 12 or 24 hours mice were transcardially perfused with PBS and a 2 mm cylindrical core sample of tissue was collected from the injection site for mRNA extraction. For immunohistochemistry, PBS perfusion was followed by 4% formaldehyde and the whole brain was processed for histology.

### qPCR for gene expression

We used a qPCR approach to quantify the relative immune gene expression and compare either between p38α^fl/fl^ and p38α^−/−^ astrocytes in response to IL-1β, IFNγ or LPS exposure, or between p38α^fl/fl^ and GFAP^cre^ p38α^−/−^ mice after intracerebral LPS injection. Total RNA was isolated and purified from either astrocytes or cylindrical core brain samples using the RNeasy lipid tissue mini kit (Qiagen). The concentration and quality of RNA was measured using a Nanodrop spectrophotometer (Nanodrop, Wilmington, DE), and the purity of RNA is verified by using the OD260/280 ratio between 1.9 and 2.1. Purified RNA (1.0 μg) was reverse-transcribed to cDNA using a Multiscribe TaqMan reverse transcription reagent kit (Applied Biosystems, Carlsbad, CA) with an oligo(dT) 15 Primer (Promega, Madison, WI). Subsequent qPCR reactions for CCL2, CCL3, CCL4, CCL5, CXCL1, CXCL2, CXCL10, IL-6, IL-1β, TNFα, COX-2, ICAM-1, VCAM-1, GFAP, Ly6G, CD11b and CD68 were performed in triplicate on a Roche Lightcycler 480 using Quantifast SYBR Green PCR kit (Qiagen) with specific primer sets listed in table S2. All experimental samples were analyzed and normalized with the expression level of Rpl13a, a selected internal control gene which is optimal for cytokine treatment studies compared to Gapdh and Sdha^49^. Either p38α^fl/fl^ astrocytes or p38α^fl/fl^ mice group at 0 hours were prepared as standard control for comparison of *in vitro* and *in vivo* immune gene expression, respectively. Relative quantification of fold-change was performed comparing Cp values (calculated by 2^nd^ derivative maximum) from individual mice by applying the 2^−ΔΔCT^ method^50^.

### Immunohistochemical staining for infiltrating cell quantification

Immunocytochemistry was performed to quantify infiltrating lymphocytes in the CNS parenchyma of p38α^fl/fl^ and GFAP^cre^p38α^−/−^ mice 24 hours after intracerebral LPS injection. Mice were anesthetized with intraperitoneal injection of ketamine (100 mg/kg) and xylazine (10 mg/kg), and then transcardially perfused with PBS followed by 4% parafomaldehyde. Brains were postfixed in 4% parafomaldehyde overnight before sectioning into 25 μm sections on a cryostat. In brief, frozen sections were incubated in blocking solution (5% goat serum with 0.03% Triton X-100) at room temperature for 1 hour, and then incubated overnight at 4°C with primary antibodies (anti-rat Iba1, Wako, Richmond, VA; anti-mouse Ly6G, BD Bioscience, San Jose, CA) prepared in the same blocking solution. Sections were washed in PBS, and then incubated in the species-appropriate fluorescent-conjugated secondary antibodies. Sections were counterstained with DAPI, and then coverslipped using Prolong Gold Anti-Fade reagent (Invitrogen). Stained sections were visualized using a Zeiss Axiophot 2 photomicroscope (Zeiss, Göttingen, Germany) and a Nikon Eclipse 2000E confocol microscope (Nikon, Melville, NY).

We quantified Ly6G-positive neutrophils in four brain sections/animal in the region closely adjacent to the LPS injection site. Due to an uneven distribution of L6G-positive cells throughout different brain regions, we selected four areas per brain section using the 10× objective of a Zeiss Axiophot 2 photomicroscope. Ly6G positive cells were visualized and counted within the selected 10× field without the examiner's knowledge of genotype. We summed the number of Ly6G-positive cells from all four areas per section to obtain the total cell number/section. The total Ly6G-positive cell number per section was averaged and statistically compared between the p38α^fl/fl^ and GFAP^cre^p38α^−/−^ genotype.

For microglia/macrophage quantification, we counted Iba1-positive cells present in the brain region near the LPS injection site. Iba1-positive cells were evenly distributed throughout the brain, thus, we counted cells from eight sections per animal adjacent to the LPS injection site. Quantification areas were selected in one area/section under 10× magnification on a Zeiss Axiophot 2 photomicroscope and Iba1-positive cells were counted with the examiner blinded to genotype. We pooled the number of cells/region and obtained an average number of Iba1-positive cells/section for each mouse. Overall data was statistically compared between the p38α^fl/fl^ and GFAP^cre^p38α^−/−^ genotype. Data are representative of three animals each for p38α^fl/fl^ and GFAP^cre^ p38α^−/−^ genotypes.

### Statistical analysis

All data analyses were performed using SigmaPlot 9.0 (San Jose, CA). Data were represented as the mean ± standard error of the mean. Each group had at least 6 mice. Statistical analysis was performed using one-way ANOVA-Tukey *post hoc* test for multiple comparisons or student *t*-test for pair-wise comparisons between p38α^fl/fl^ and GFAP^cre^p38α^−/−^ genotypes at the same time point. A value of p ≤ 0.05 was considered statistically significant.

## Results

### GFAP^cre^p38α^−/−^ mice were viable with no phenotypic abnormalities

**Figure 1:**
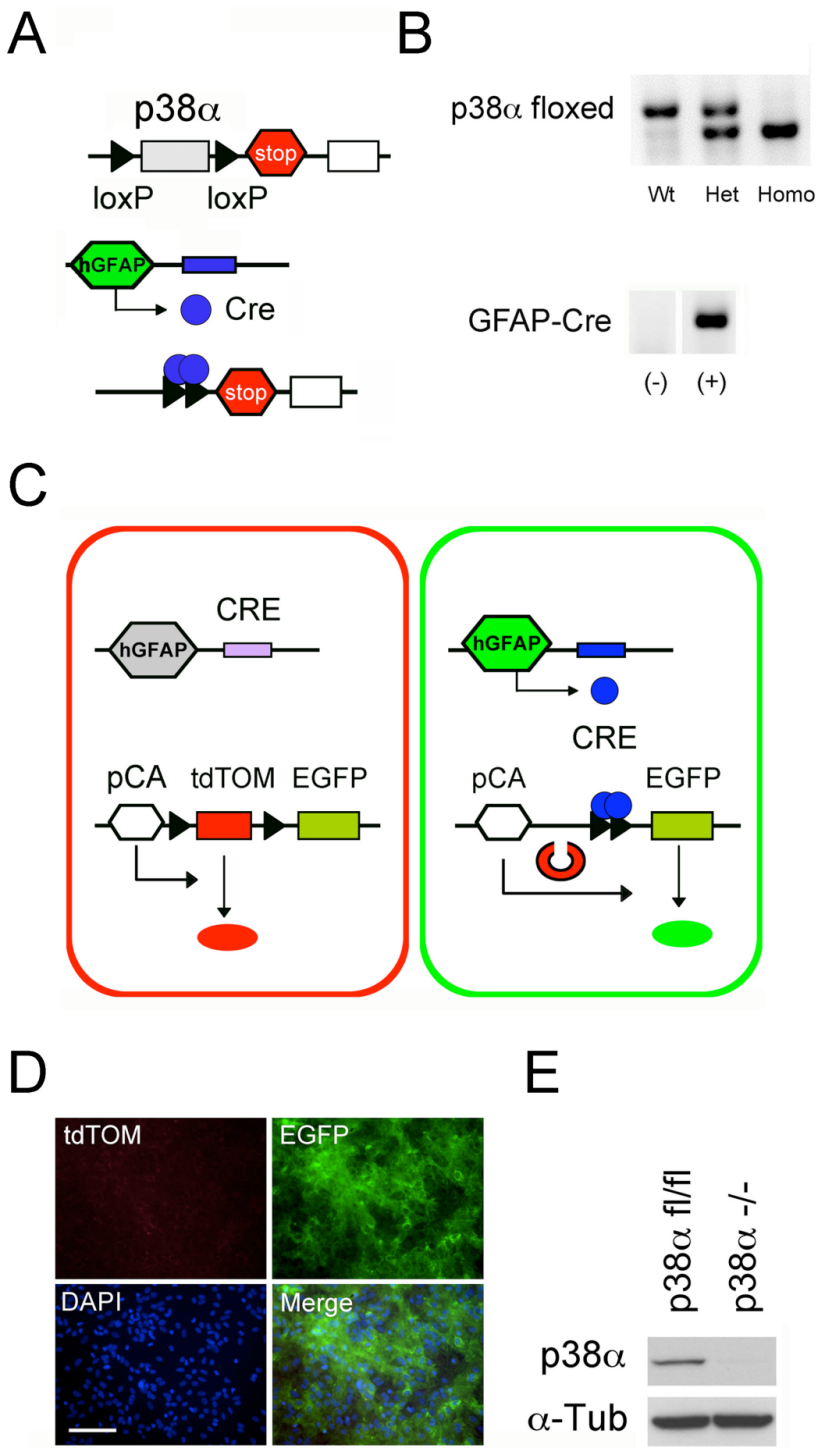
Generation of GFAP^Cre^p38α^−/−^ mice and establishment of highly purified p38α^−/−^ astrocyte cultures. (A) GFAP^Cre^p38α^−/−^ mice were generated by crossing hGFAP-Cre mice and mice with p38α-floxed alleles. Schematic shows the targeted genetic locus of p38α gene flanked by two loxP sites. Deletion of p38α gene is driven by Cre-loxP recombination that is under the control of hGFAP promoter activity in cells. (B) Genotyping of GFAP^cre^p38α^−/−^ mice demonstrating homozygosity of p38α-floxed allele and positive expression of hGFAP-Cre transgene. (C) Schematic showing the ROSA26tdTomato reporter system. Under the control of hGFAP promoter, Cre recombination occurs leads to excision of tdTomato(mT) and expression of EGFP(mG) in GFAP-positive cells. This results in the change of membrane-bound red fluorescence (tdTomato) to a membrane-bound green fluorescence (EGFP) in cells. (D) Pure astrocyte cultures established from pups from cross breeds of ROSA26-tdTomato(mT)/EGFP(mG) reporter line and hGFAP-Cre mice. The purity of these astrocyte cultures was verified by the predominance of EGFP-positive astrocytes, with rare occurrence of tdTomato-positive cells (hGFAP-Cre negative; scale bar = 100 μm). (F) In comparison to astrocyte cultures derived from p38α^fl/fl^ mice, western blot analysis confirmed that the p38α protein is absent in p38α^−/−^ astrocyte cultures.

To study the specific role of p38α in astrocytes, we generated a conditional GFAP-specific p38α knockout mouse by crossing hGFAP-Cre [FVB-Tg(GFAP-cre)25Mes/J] mice with p38α-floxed [B6.129-Mapk14<tm1.2Otsu>] mice. The targeted disruption of p38α gene in GFAP^cre^p38α^−/−^ mice is mediated by Cre-loxP recombination under the control of hGFAP promoter, and p38α gene deletion only occurred in specific cell types with an active hGFAP promoter as illustrated (Fig. 1A). The resulting genotype of GFAP^cre^p38α^−/−^ mice was detected by the presence of homozygous p38α-floxed alleles and being positive for hGFAP-Cre transgene (Fig. 1B). The homozygous p38α^fl/fl^ mice without hGFAP-Cre were indistinguishable from the GFAP^cre^p38α^−/−^ littermates in phenotypic appearance, behavior, and body weights. Histological examination also did not show any astrocyte-specific developmental defects in the central nervous system. In addition to studying the *in vivo* effects of p38α in astrocytes during CNS inflammation, the generation of these mice also allowed us to establish p38α^fl/fl^ and p38α^−/−^ astrocyte cultures. Using these cultures, we could examine the immune activity of astrocytes in the absence of other immune cells and the complex inflammatory milieu seen in the *in vivo* CNS.

### Pure astrocyte cultures could be established from both p38α^−/−^ and p38α^fl/fl^ astrocytes

We first generated astrocyte cultures using pups from cross breed of homozygous ROSA26-tdTomato(mT)-EGFP(mG) and hGFAP-Cre mice, in which, tdTomato gene is excised by Cre recombinase, leading to EGFP expression in cells with an active hGFAP promoter (Fig. 1C). We then confirmed the purity of astrocyte cultures using this double fluorescent reporter cells in primary culture. After expansion of these cultures, we identified a confluent population of GFAP^+^ astrocytes expressing EGFP that were predominant with no tdTomato positive cells indicating the absence of contaminating cell types that would include microglia, oligodendrocytes, and fibroblasts (Fig 1D). This protocol could be consistently reproduced and was used to derive highly pure populations of astrocytes that could be used for subsequent *in vitro* experiments. After astrocyte cultures were made from p38α^fl/fl^ and GFAP^cre^p38α^−/−^ mice, we also verified for the absence of p38α protein expression in p38α^−/−^ cultures (Fig 1E).

### Altered JNK, and ERK 1/2 signaling in IL-1β activated p38α^−/−^ astrocytes

**Figure 2:**
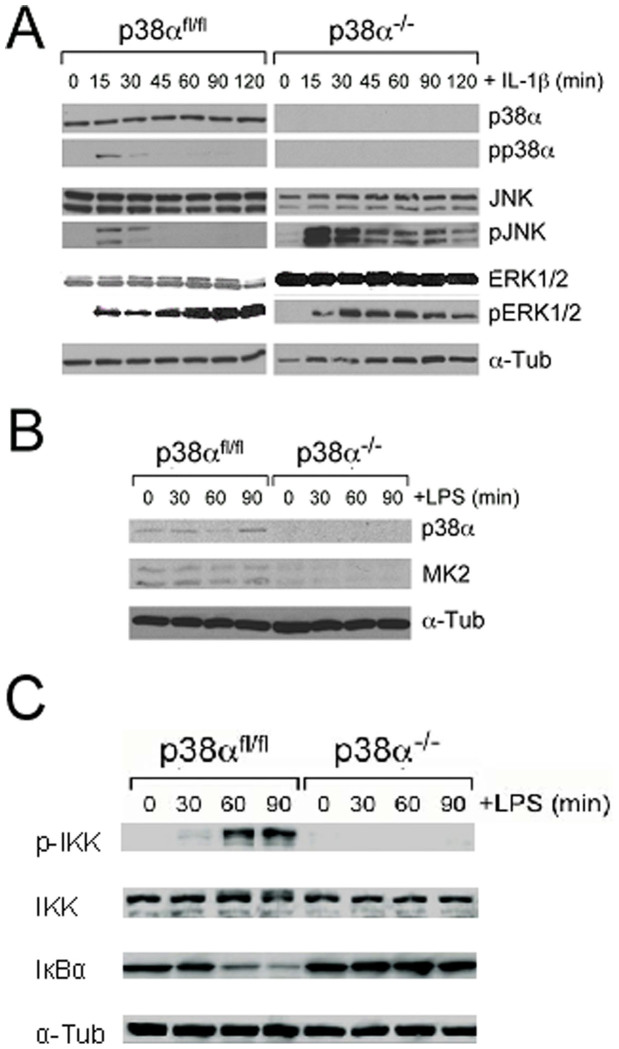
Western blot analysis of p38, JNK, ERK1/2 MAPK, MK2 and NF-κB signaling in p38α^−/−^ and p38α^fl/fl^ astrocytes. (A) MAPK signaling in p38α^−/−^ astrocytes was examined with western blot analysis after IL-1β treatment. Exposure to IL-1β led to an acute and enhanced activation of JNK phosphorylation in p38α^−/−^ compared to p38α^fl/fl^ astrocytes. Although phosphorylated ERK1/2 was not found in resting p38α^−/−^ astrocytes, IL-1β stimulation resulted in ERK1/2 phosphorylation over time but this phosphorylation remained at lower levels compared to p38α^fl/fl^ controls. (B) The MK2 activation in p38α^−/−^ astrocytes was examined with western blot analysis after LPS treatment. Exposure to LPS stimulation showed reduced expression of MK2 in p38α^−/−^ astrocytes compared to p38α^fl/fl^ astrocytes. (C) The NF-κB activity was examined in p38α^−/−^ astrocytes compared to p38α^fl/fl^ controls in response to LPS stimulation. Immunoblot analysis showed that immune activation by LPS treatment did not induce phosphorylated IκB kinase (p-IKK) protein in p38α^−/−^ astrocytes compared to p38α^fl/fl^ astrocytes and did not induce degradation of IκBα protein in p38α^−/−^ astrocytes compared to p38α^fl/fl^ controls. The gels have been run under the same experimental conditions.

Cross talk between MAPK signalling pathways commonly occurs during cellular response to inflammatory stress. We examined whether p38α deficiency in astrocytes affects JNK and ERK1/2 activation in response to IL-1β stimulation. We observed an early phosphorylation of JNK and ERK1/2 proteins in both p38α^fl/fl^ and p38α^−/−^ astrocytes at 15 min after IL-1β treatment. This indicates that p38α deficiency in astrocytes has a minimal effect on initiating ERK1/2 and JNK signaling. Although phosphorylated ERK1/2 was not found in resting p38α^−/−^ astrocytes, IL-1β stimulation resulted in a sustained ERK1/2 phosphorylation over time in both p38α^fl/fl^ and p38α^−/−^ astrocytes. Moreover, ERK1/2 phosphorylation remained at lower levels in p38α^−/−^ astrocytes compared to p38α^fl/fl^ controls. In contrast, we observed a relatively enhanced and sustained JNK activation in p38α^−/−^ astrocytes indicated by increased phosphorylation levels over the entire duration of IL-1β exposure compared to an acute drop off of JNK phosphorylation in p38α^fl/fl^ astrocytes (Fig. 2A). This suggests that the JNK pathway is highly activated by IL-1β in the absence of p38α signaling in astrocytes.

### Reduced MK2 protein levels in p38α^−/−^ astrocytes in response to LPS

Studies have shown that p38 MAPKs regulate inflammatory gene expression through an MK2-dependent AU-rich element (ARE)-containing mRNA stabilization mechanism^51^. We examined whether p38α deficiency has an effect on MK2 expression in astrocytes before and during immune activation using LPS. In this study, we observed that basal levels of MK2 protein in p38α^−/−^ astrocytes were undetectable in a western blot compared to p38α^fl/fl^ astrocytes (Fig. 2B). In a time course, immune activation by LPS treatment did not induce MK2 expression in p38α^−/−^ astrocytes. Basal levels of MK2 expression observed in p38α^fl/fl^ astrocytes were also unaffected by LPS treatment. These data suggest that p38α deficiency has a negative effect on MK2 expression in astrocytes.

### Diminished NF-κB activity in p38α^−/−^ astrocytes in response to LPS

We next investigated whether p38α deficiency has an effect on the NF-κB activity in the p38α^−/−^ or p38α^fl/fl^ astrocytes after LPS stimulation. By immunoblot analysis, we observed that immune activation by LPS treatment did not induce phosphorylated IκB kinase (p-IKK) protein in p38α^−/−^ astrocytes compared to p38α^fl/fl^ astrocytes (Fig. 2C). In the meanwhile, LPS treatment did not induce degradation of IκBα protein in p38α^−/−^ astrocytes (Fig. 2C). These data suggest that NF-κB is involved in p38α regulation of pro-inflammatory cytokine and chemokine expression in astrocytes.

### LPS induces differential cytokine, chemokine and adhesion molecule expression in p38α^−/−^ and p38α^fl/fl^ astrocytes

**Figure 3:**
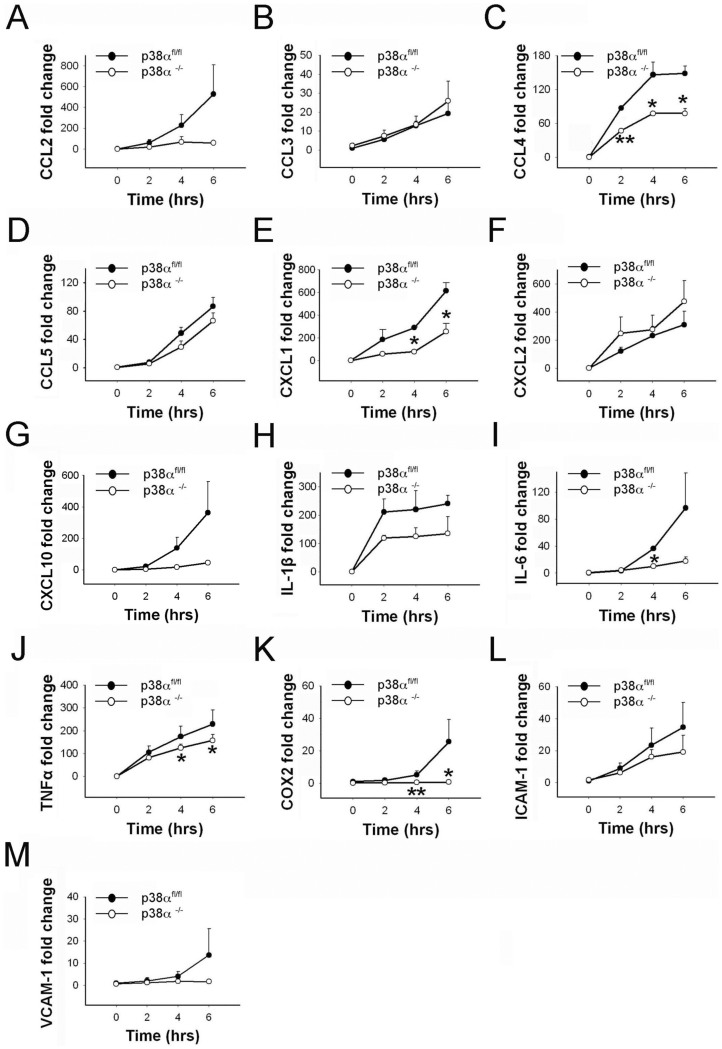
Cytokine, chemokine and cell adhesion molecule expression in p38α^−/−^ astrocytes as compared to p38α^fl/fl^ astrocytes with LPS treatment. (A) CCL2. (B) CCL3. (C) CCL4. (D) CCL5. (E) CXCL1. (F) CXCL2. (G) CXCL10. (H) IL-1β. (I) IL-6. (J) TNFα. (K) COX-2. (L) ICAM-1. (M) VCAM-1. *p < 0.05 and **p < 0.01; data represent mean ± SEM.

We examined the production of different chemokines CCL2, CCL3, CCL4, CCL5, CXCL1, CXCL2 and CXCL10 in p38α^−/−^ and p38α^fl/fl^ astrocytes in response to LPS exposure. CCL2 and CXCL10 mRNA levels were significantly upregulated over time after LPS treatments in control p38α^fl/fl^ astrocytes (p < 0.05). In contrast, a relatively moderate but significant change in CXCL10 expression was seen in p38α^−/−^ astrocytes over time after LPS treatment (p < 0.05; Fig. 3G), whereas a moderate but significant change of CCL2 expression was only detected in p38α^−/−^ astrocytes at 6 hours after LPS treatment (p < 0.05; Fig. 3A). However, this difference in trend of CCL2 and CXCL10 mRNA expression did not reach significance when statistically compared between the two genotypes. On the other hand, both CCL4 and CXCL1 mRNA levels were significantly upregulated in p38α^fl/fl^ and p38α^−/−^ astrocytes response to LPS exposure (p < 0.05). In particular, significantly lower expression of CCL4 was observed in p38α^−/−^ astrocytes at 2, 4 and 6 hours after LPS treatment, compared to p38α^fl/fl^ astrocytes (p < 0.05; Fig. 3C). CXCL1 mRNA expression was significantly lower in p38α^−/−^ astrocytes compared to p38α^fl/fl^ astrocytes at 4 and 6 hours after LPS treatment (p < 0.05; Fig. 3E). On a moderate scale, a significant upregulation of CCL3, CCL5, and CXCL2 mRNA expression was seen in both p38α^−/−^ and p38α^fl/fl^ astrocytes over time after LPS treatment (p < 0.05) with no difference between the genotypes for any of the time points (Fig. 3B, D and F).

We then examined the expression of IL-6, IL-1β, TNFα, and COX-2 in p38α^−/−^ and p38α^fl/fl^ astrocytes after LPS treatment. Expression of IL-1β mRNA level was significantly upregulated and sustained throughout the duration of LPS treatment in both p38α^−/−^ and p38α^fl/fl^ astrocytes (p < 0.05; Fig. 3H). We found no statistical differences between the two genotypes at any of the three time points for LPS treatment. A significant upregulation of IL-6 mRNA was observed in control p38α^fl/fl^ astrocytes over time after LPS exposure (p < 0.05). Only a moderate but significant upregulation of IL-6 was seen in p38α^−/−^ astrocytes over time after LPS treatment (p < 0.05). IL-6 mRNA expression was significantly lower in p38α^−/−^ astrocytes compared to p38α^fl/fl^ astrocytes at 4 hours after LPS treatment (p < 0.05; Fig. 3I). There was significant upregulation of TNFα mRNA expression in both p38α^−/−^ and p38α^fl/fl^ astrocytes in response to LPS (p < 0.05). TNFα mRNA expression in p38α^−/−^ astrocytes was significantly lower compared to p38α^fl/fl^ astrocytes at 4 and 6 hours after LPS treatment (p < 0.05; Fig. 3J). In response to LPS, there was a moderate upregulation of COX-2 mRNA seen in p38α^fl/fl^ astrocytes, whereas no distinguishable change of COX-2 mRNA levels was observed in p38α^−/−^ astrocytes. COX-2 mRNA expression was significantly lower in p38α^−/−^ astrocytes compared to p38α^fl/fl^ astrocytes at 4 and 6 hours after LPS treatment (p < 0.05; Fig. 3K). This result suggests that p38α plays a predominant role in regulating IL-6, TNFα and COX-2 expression in LPS-stimulated astrocytes.

We also examined whether p38α regulates the induction of ICAM-1 and VCAM-1 in astrocytes after LPS exposure. ICAM-1 showed a trend of upregulation but did not reach statistical significance for both p38α^fl/fl^ and p38α^−/−^ astrocytes when compared at different time points after LPS exposure (Fig. 3L). Similarly, for VCAM-1, we did not observe any significant differences in mRNA levels after LPS exposure (Fig. 3M). We also did not detect any statistically significant differences for ICAM-1 and VCAM-1 expression levels between p38α^−/−^ and p38α^fl/fl^ astrocytes after LPS treatment.

### Interleukin-1β induces differential cytokine, chemokine and adhesion molecule expression in p38α^−/−^ and p38α^fl/fl^ astrocytes

**Figure 4:**
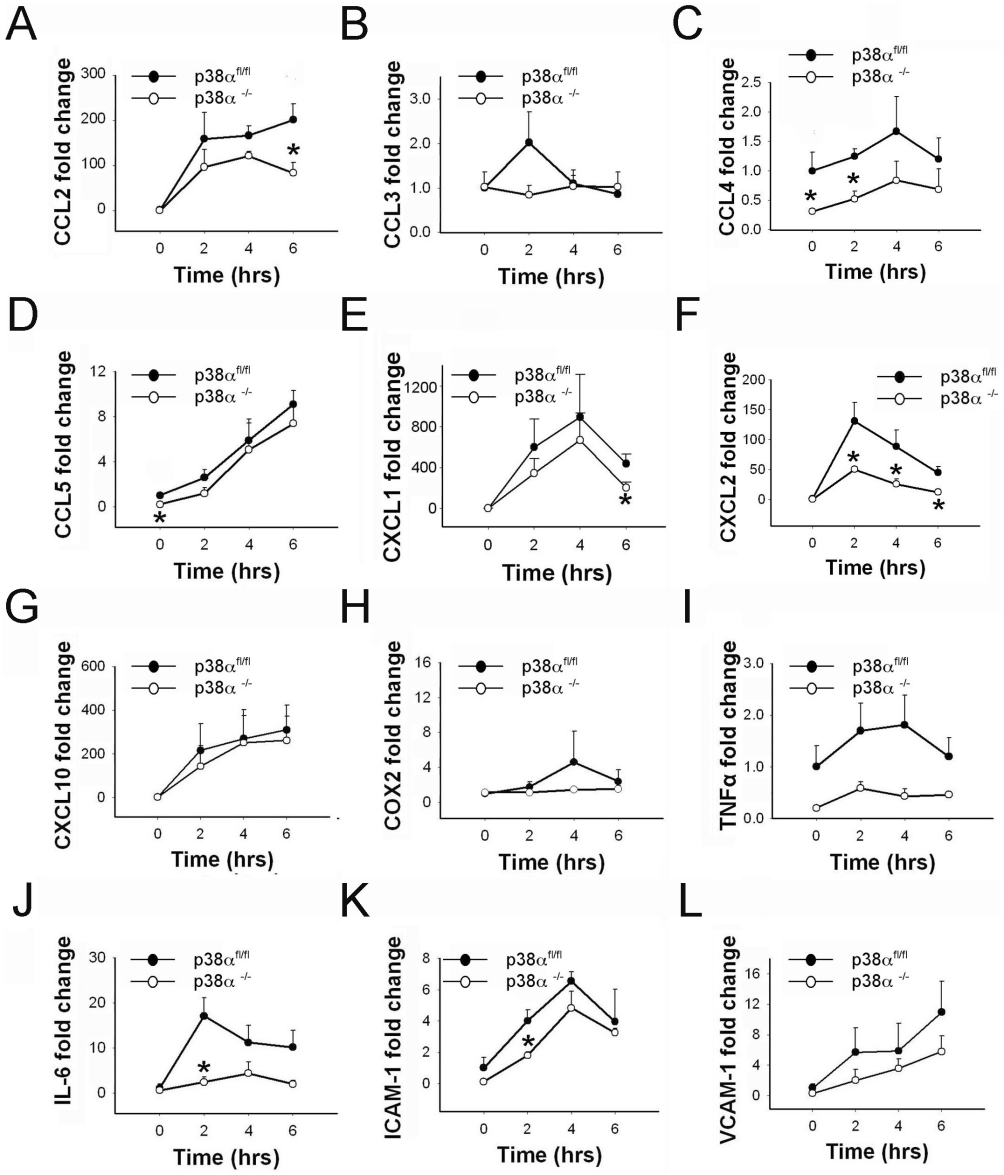
Cytokine, chemokine and cell adhesion molecule expression in p38α^−/−^ astrocytes as compared to p38α^fl/fl^ astrocytes with IL-1β treatment. (A) CCL2. (B) CCL3. (C) CCL4. (D) CCL5. (E) CXCL1. (F) CXCL2. (G) CXCL10. (H) COX-2. (I) TNFα. (J) IL-6. (K) ICAM-1. (L) VCAM-1. *p < 0.05; data represent mean ± SEM.

We examined the specific effects of IL-1β on the activation of chemokines CCL2, CCL3, CCL4, CCL5, CXCL1, CXCL2, and CXCL10 in p38α^fl/fl^ and p38α^−/−^ astrocytes. CCL2 and CXCL1 mRNA levels were significantly upregulated in both p38α^fl/fl^ and p38α^−/−^ astrocytes after IL-1β treatment (p < 0.05; Figure 4A and E). However, CCL2 expression was significantly lower in p38α^−/−^ astrocytes at 6 hours compared to p38α^fl/fl^ astrocytes after IL-1β treatment. (p < 0.05; Fig. 4A). After an upregulation at time points 2 and 4 hours, CXCL1 mRNA levels dropped in both genotypes at 6 hours after IL-1β exposure; we found that this decrease in CXCL1 expression was significantly lower in p38α^−/−^ astrocytes compared to p38α^fl/fl^ astrocytes (p < 0.05; Fig. 4E). A significant short-term upregulation of CXCL2 was detected in both p38α^−/−^ and p38α^fl/fl^ astrocytes after IL-1β treatment (p < 0.05). However, we detected that CXCL2 expression was significantly lower in p38α^−/−^ astrocytes at time points 2, 4 and 6 hours after IL-1β exposure compared to p38α^fl/fl^ astrocytes (p < 0.05; Fig. 4F). CCL3 mRNA levels remained unchanged after IL-1β treatment in both p38α^−/−^ and p38α^fl/fl^ astrocytes (Fig. 4B). Interestingly, we found significantly lower basal levels of CCL4 in p38α^−/−^ and p38α^fl/fl^ astrocytes. After IL-1β exposure, CCL4 appeared to be upregulated at 2 and 4 hours but was not statistically significant. Even after 2 hours of IL-1β treatment, CCL4 in p38α^−/−^ astrocytes was significantly lower than p38α^fl/fl^ astrocytes (p < 0.05; Fig. 4C). A moderate but significant linear upregulation of CCL5 mRNA expression was seen in both p38α^−/−^ and p38α^fl/fl^ astrocytes over time after IL-1β treatment (p < 0.05). However, we did not detect significant differences between the genotypes for any of the time points (Fig. 4D). CXCL10 expression showed a similar trend of upregulation but only reached statistical significance in both p38α^fl/fl^ and p38α^−/−^ astrocytes at 6 hours after IL-1β exposure compared to 0 hours (p < 0.05; Fig 4G). We did not detect any statistically significant differences for CXCL10 expression levels between p38α^−/−^ and p38α^fl/fl^ astrocytes after IL-1β treatment (Fig. 4G).

In addition to chemokines, we examined whether p38α has a regulatory effect on IL-1β-induced COX-2 activation in astrocytes. Our results showed no differences in COX-2 mRNA expression either temporal or between p38α^−/−^ and p38α^fl/fl^ astrocytes in response to IL-1β treatment (Fig. 4H). We also examined for the activation of other inflammatory mediators such as TNFα and IL-6 in p38α^fl/fl^ and p38α^−/−^ astrocytes after IL-1β exposure. IL-6 mRNA levels were significantly upregulated after 2-, 4- and 6-hour IL-1β treatments in control p38α^fl/fl^ astrocytes (p < 0.05). In contrast, no significant change in IL-6 expression was seen in p38α^−/−^ astrocytes at these time points after IL-1β treatment. In particular, we detected a significantly lower expression of IL-6 in p38α^−/−^ astrocytes at 2 and 6 hours after IL-1β exposure compared to p38α^fl/fl^ astrocytes (p < 0.05; Fig. 4J). We found no significant differences in TNFα mRNA expression over time or between p38α^fl/fl^ and p38α^−/−^ astrocytes in response to IL-1β exposure (Fig. 4I).

We next examined for the effect of IL-1β treatment on ICAM-1 and VCAM-1 expression in astrocytes. We found moderate but significant upregulation of ICAM-1 in both p38α^fl/fl^ and p38α^−/−^ astrocytes over time after IL-1β treatment that peaked at 4 hours (p < 0.05). ICAM-1 levels in p38α^−/−^ astrocytes at 2 hours after IL-1β treatment was significantly lower compared to p38α^fl/fl^ astrocytes (p < 0.05; Fig. 4K). Similarly, VCAM-1 showed a trend of upregulation for the entire duration of IL-1β exposure but did not reach statistical significance for both p38α^fl/fl^ and p38α^−/−^ astrocytes. We also did not observe any significant differences in VCAM-1 mRNA levels between genotypes at the three time points after IL-1β exposure (Fig. 4L). These data suggest that p38α plays a crucial role during early induction of ICAM-1 in astrocytes after IL-1β exposure.

### Interferon γ induces differential cytokine, chemokine and adhesion molecule mRNA expression in p38α^−/−^ and p38α^fl/fl^ astrocytes

**Figure 5:**
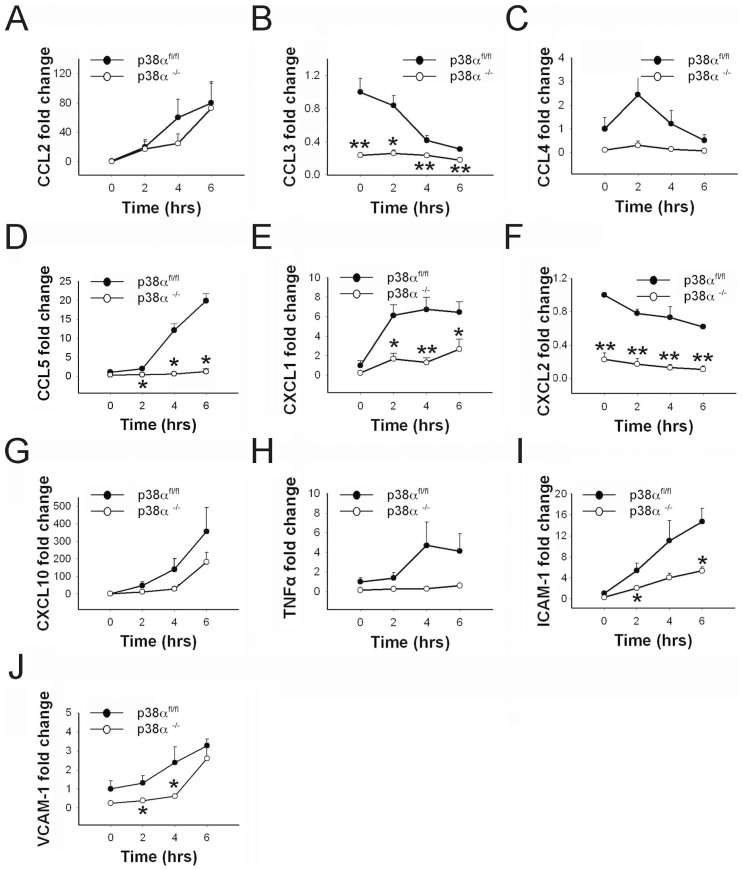
Cytokine, chemokine and cell adhesion molecule expression in p38α^−/−^ astrocytes as compared to p38α^fl/fl^ astrocytes with IFNγ treatment. (A) CCL2. (B) CCL3. (C) CCL4. (D) CCL5. (E) CXCL1. (F) CXCL2. (G) CXCL10. (H) TNFα. (I) ICAM-1. (J) VCAM-1. *p < 0.05; data represent mean ± SEM.

We examined whether IFNγ-induced activation of astrocytes regulates CCL2, CCL3, CCL4, CCL5, CXCL1, CXCL2, and CXCL10 gene expression through the p38α signaling pathway. Our results show that a significant upregulation of CCL2 mRNA expression was seen in p38α^fl/fl^ astrocytes after IFNγ treatment (p < 0.05), whereas in p38α^−/−^ astrocytes a significant upregulation of CCL2 was detected only at 6 hours after IFNγ treatment (p < 0.05; Fig 5A). Similarly, CXCL10 mRNA levels were significantly upregulated after 2-, 4- and 6-hour IFNγ treatments in control p38α^fl/fl^ astrocytes (p < 0.05), whereas a relatively moderate but significant change in CXCL10 expression was seen in p38α^−/−^ astrocytes only at 4 and 6 hours after IFNγ treatment (p < 0.05; Fig 5G). However, these trends of CCL2 and CXCL10 mRNA expression did not reach statistical significance when compared between the two genotypes (Fig. 5A and G). For CCL5, mRNA levels were significantly upregulated at 4 and 6 hours after IFNγ treatment in control p38α^fl/fl^ astrocytes (p < 0.05), whereas no significant change was observed in p38α^−/−^ astrocytes over time after IFNγ treatment suggesting that CCL5 expression was refractory to IFNγ treatment in p38α^−/−^ astrocytes. Supporting this observation, statistics comparing p38α^−/−^ astrocytes at 2, 4 and 6 hours to p38α^fl/fl^ astrocytes after IFNγ exposure showed significant differences between the two genotypes (p < 0.05; Fig. 5D). Similarly, CXCL1 expression increased significantly in p38α^fl/fl^ astrocytes after IFNγ treatment (p < 0.05), but only moderately significant fold-changes in the expression of CXCL1 were seen in p38α^−/−^ astrocytes (p < 0.05). However, CXCL1 expression was significantly lower in p38α^−/−^ astrocytes at 2, 4 and 6 hours compared to p38α^fl/fl^ astrocytes (p < 0.05; Fig. 5E). Baseline levels of CCL3 and CXCL2 were significantly lower in p38α^−/−^ astrocytes compared to p38α^fl/fl^ astrocytes (p < 0.05). In response to IFNγ treatment, CCL3 and CXCL2 levels were significantly downregulated in p38α^fl/fl^ astrocytes; but there were minimal to no effects on p38α^−/−^ astrocytes. Pairwise comparisons between the two genotypes fold-change values remained significantly different for 0, 2, 4 and 6 hours after IFNγ exposure (p < 0.05; Fig. 5B and 5F). Similar to trends observed for CCL3 and CXCL2, we found lower baseline expression of CCL4 in p38α^−/−^ astrocytes and no observable upregulation in this genotype. Although there was upregulation of CCL4 in p38α^fl/fl^ astrocytes after IFNγ treatment, this difference did not reach statistical significance. Despite this general trend, we did not detect any statistically significant differences for CCL4 expression in comparisons between p38α^−/−^ and p38α^fl/fl^ astrocytes (Fig. 5C).

We also examined whether p38α regulates TNFα expression during an IFNγ-induced astrocytic response. A moderate upregulation of TNFα was observed in p38α^fl/fl^ astrocytes at 4 and 6 hours after IFNγ exposure, whereas in p38α^−/−^ astrocytes, TNFα expression continued to remain at baseline levels even at 2, 4 and 6 hours after IFNγ treatment. However, this difference in trend of TNFα mRNA fold-change did not reach statistical significance when compared between the two genotypes (Fig. 5H).

We next examined the role of p38α in regulating adhesion molecule expression in astrocytes induced by IFNγ. We observed a moderate but significant upregulation of both ICAM-1 and VCAM-1 in p38α^fl/fl^ and p38α^−/−^ astrocytes response to IFNγ exposure (p < 0.05). However, we noted significantly lower upregulation of ICAM-1 in p38α^−/−^ astrocytes at 2 and 6 hours after IFNγ treatment compared to p38α^fl/fl^ astrocytes (p < 0.05; Fig. 5I). Similarly, a significantly lower upregulation of VCAM-1 was seen in p38α^−/−^ astrocytes at 2 and 4 hours after IFNγ exposure compared to p38α^fl/fl^ astrocytes (p < 0.05; Fig. 5J). Overall, this data suggests that p38α plays an essential role in regulating CCL5, CXCL1, ICAM-1 and VCAM-1 activation in astrocytes exposed to IFNγ treatment, while having a minimal effect on CCL2, CXCL10 and TNFα induction.

### Intracerebral LPS injection induces differential cytokine, chemokine and adhesion molecule expression in GFAP^cre^p38α^−/−^ mice

**Figure 6:**
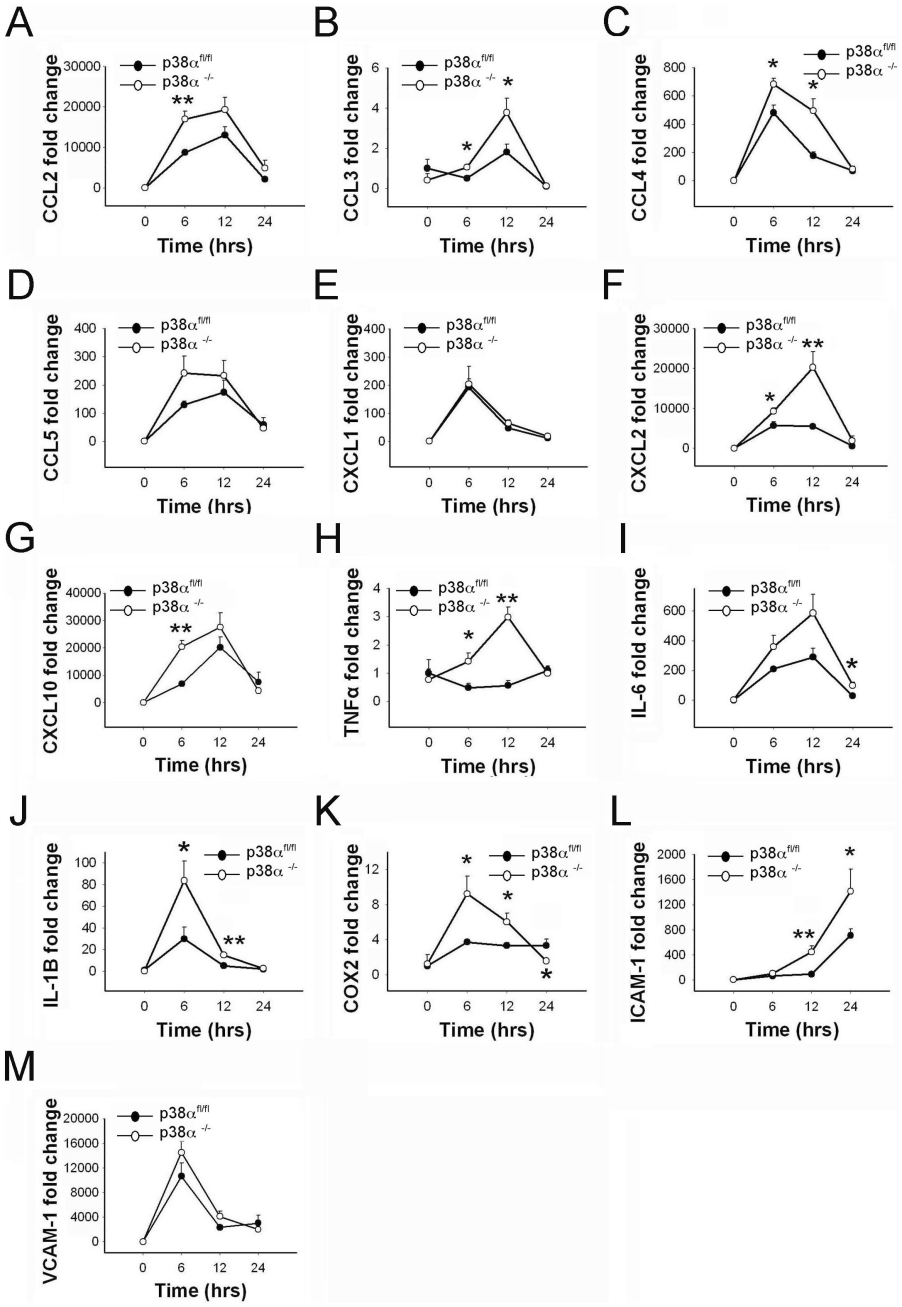
Chemokine, cytokine and cell adhesion molecule expression in the brain of GFAP^Cre^ p38α^−/−^ mice as compared to p38α^fl/fl^ mice after intracerebral LPS injection. (A) CCL2. (B) CCL3. (C) CCL4. (D) CCL5. (E) CXCL1. (F) CXCL2. (G) CXCL10. (H) TNFα. (I) IL-6. (J) IL-1β. (K) COX-2. (L) ICAM-1. (M) VCAM-1. *p < 0.05 and **p < 0.01; data represent mean ± SEM.

We examined the induction of several specific chemokines. CCL2, and CXCL10 mRNA levels were significantly upregulated in p38α^fl/fl^ and GFAP^cre^p38α^−/−^ mice at 6 and 12 hours after intracerebral LPS injection (p < 0.05). In comparison between the two genotypes, we found a significantly higher expression of CCL2 and CXCL10 in GFAP^cre^p38α^−/−^ mice at 6 hours after LPS injection (p < 0.05; Fig. 6A and 6G). For CCL3, a moderate but significant upregulation was observed in GFAP^cre^p38α^−/−^ mice at 6 and 12 hours after intracerebral LPS injection (p < 0.05), while no observable changes of CCL3 were seen in p38α^fl/fl^ cohorts at these time points. Therefore, levels of CCL3 were also significantly higher in GFAP^cre^p38α^−/−^ mice at 6 and 12 hours after intracerebral LPS injection (p < 0.05; Fig. 6B). Similarly, CXCL2 mRNA levels were significantly upregulated at 6 and 12 hours in GFAP^cre^p38α^−/−^ mice after intracerebral LPS injection (p < 0.05). Although upregulation was moderate in p38α^fl/fl^ cohorts it was also significant at 6 and 12 hours compared to 0 hours. Comparing CXCL2 expression between the two genotypes showed a significantly higher expression in GFAP^cre^p38α^−/−^ mice at 6 and 12 hours compared to p38α^fl/fl^ cohorts (p < 0.05; Fig. 6F). For CCL4, significant upregulation was observed in both GFAP^cre^p38α^−/−^ mice and p38α^fl/fl^ mice after LPS injections at 6 and 12 hours. When expression levels were compared between the two genotypes, we found CCL4 expression to be significantly higher in GFAP^cre^p38α^−/−^ mice at both 6 and 12 hours compared to p38α^fl/fl^ cohorts (p < 0.05; Fig. 6C). Expression of CCL5 was significantly upregulated in both p38α^fl/fl^ and GFAP^cre^p38α^−/−^ mice in the time course (6 and 12 hours) after intracerebral LPS injection (p < 0.05). We found no statistical differences between the two genotypes for CCL5 at any of the three time points (Fig. 6D). Similarly, expression of CXCL1 was significantly upregulated in both p38α^fl/fl^ and GFAP^cre^p38α^−/−^ mice at 6 and 12 hours after intracerebral LPS injection (p < 0.05); we detected no statistically significant differences in CXCL1 levels between the genotypes (Fig. 6E).

In addition to chemokines, we also examined the expression of TNFα, IL-6, and IL-1β in p38α^fl/fl^ and GFAP^cre^p38α^−/−^ mice after intracerebral LPS injections. We found a moderate but significant upregulation of TNFα mRNA in GFAP^cre^p38α^−/−^ mice at 12 hours after intracerebral LPS injection (p < 0.05), whereas no observable upregulation of TNFα mRNA level were detected in p38α^fl/fl^ cohorts over time after LPS injection. Differences were significant with higher fold-change in GFAP^cre^p38α^−/−^ mice compared to p38α^fl/fl^ cohorts at 6 and 12 hours after LPS injections (p < 0.05; Fig. 6H). We observed significant upregulation of IL-6 in both p38α^fl/fl^ and GFAP^cre^p38α^−/−^ mice over time after LPS injection (p < 0.05). Although no differences were observed between genotypes at the peak of IL-6 expression at 12 hours after LPS injection, a significantly higher expression of IL-6 was detected in GFAP^cre^p38α^−/−^ mice at 24 hours after LPS injection, compared to p38α^fl/fl^ cohorts (p < 0.05; Fig. 6I). On the other hand, IL-1β mRNA levels were significantly upregulated in GFAP^cre^p38α^−/−^ mice over time after LPS injection (p < 0.05). In contrast, a relatively moderate change in IL-1β expression was seen in p38α^fl/fl^ cohorts at 6 and 12 hours after LPS injection (p < 0.05). In particular, a significantly higher expression of IL-1β was observed in GFAP^cre^p38α^−/−^ mice at 6 and 12 hours after LPS injection, compared to p38α^fl/fl^ cohorts (p < 0.05; Fig. 6J). *In vivo* evidence indicated that systemic LPS injection stimulates COX-2 production within the CNS microvaculature^52^. We thus examined the COX-2 expression in p38α^fl/fl^ and GFAP^cre^p38α^−/−^ mice following intracerebral LPS injection. A significant upregulation of COX-2 mRNA was observed in GFAP^cre^p38α^−/−^ mice at 6 and 12 hours after LPS injection (p < 0.05), while a moderate but significant upregulation of COX-2 was seen in p38α^fl/fl^ cohorts over time after LPS injection (p < 0.05). In particular, we observed a significantly higher COX-2 mRNA expression in GFAP^cre^p38α^−/−^ mice at 6 and 12 hours after LPS injection, compared to p38α^fl/fl^ cohorts (p < 0.05; Fig. 6K). Interestingly, COX-2 expression levels returned to baseline at 24 hours in the GFAP^cre^p38α^−/−^ mice, and were significantly lower compared to p38α^fl/fl^ cohorts at this time point (p < 0.05). Moreover, ICAM-1 and VCAM-1 are crucial adhesion molecules expressed by cellular components of CNS microvasculature including perivascular astrocytes^53^. Therefore, we examined the induction of VCAM-1 and ICAM-1 in p38α^fl/fl^ and GFAP^cre^p38α^−/−^mice following intracerebral LPS injection. ICAM-1 mRNA levels were significantly upregulated in p38α^fl/fl^ and GFAP^cre^p38α^−/−^ mice at 12 and 24 hours after intracerebral LPS injection (p < 0.05). Particularly, in comparison with p38α^fl/fl^ cohorts, a significantly higher expression of ICAM-1 was observed in GFAP^cre^p38α^−/−^ mice at 12 and 24 hours after LPS injection (p < 0.05; Fig. 6L). In contrast, a significant upregulation of VCAM-1 mRNA expression was seen in both p38α^fl/fl^ and GFAP^cre^p38α^−/−^ mice over time and peaked at 6 hours after intracerebral LPS injection (p < 0.05). However, we did not detect significant differences of VCAM-1 levels between the genotypes for any of the time points after LPS injection (Fig. 6M). Therefore, astrocyte p38α signaling modulates *in vivo* ICAM-1 expression but not VCAM-1 expression during an intracerebral LPS challenge. Taken together, *in vivo* findings identified significantly higher expression of specific subsets of chemokines, cytokines, and adhesion molecules in the CNS of GFAP^cre^ p38α^−/−^ mice challenged with intracerebral LPS injection.

**Figure 7:**
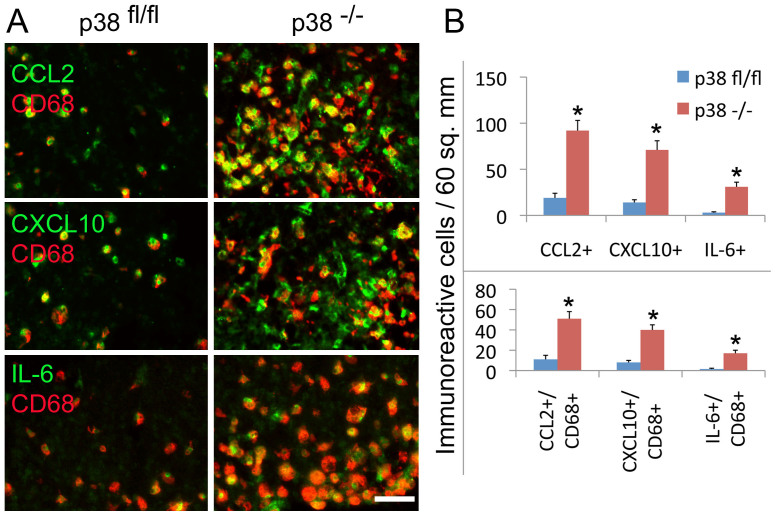
Immunostaining and quantification of chemokine and cytokine protein expression in the brain of GFAP^Cre^ p38α^−/−^ and p38α^fl/fl^ mice after intracerebral LPS injection. (A) The immunoreactivity of CCL2, CXCL10 and IL-6 was observed in the increased population of CD68+ cells in the brain of GFAP^cre^ p38α^−/−^ mice compared to p38α^fl/fl^ mice at 6 hours after LPS stimulation. (B) Quantification of immunoreactive cells expressing CCL2, CXCL10 and IL-6 and the expression of CCL2, CXCL10 and IL-6 in the population of CD68+ cells in the brain of GFAP^cre^ p38α^−/−^ mice compared to p38α^fl/fl^ mice at 6 hours after LPS stimulation.

To corroborate these results based on mRNA levels using an alternative approach by immunohistochemical analysis of cytokine and chemokine expression, we performed and verified the immunoreactivity of CCL2, CXCL10 and IL-6 in the increased population of CD68+ cells in the brain of GFAP^cre^ p38α^−/−^ mice compared to p38α^fl/fl^ mice at 6 hours after LPS challenge (Fig. 7A). Quantification of immunoreactive cells expressing CCL2, CXCL10 and IL-6 showed the increased expression of CCL2, CXCL10 and IL-6 and particularly their expression in the increased population of CD68+ cells in the brain of GFAP^cre^ p38α^−/−^ mice compared to p38α^fl/fl^ mice after LPS stimulation (Fig. 7B).

### Reduced astrocyte activation in GFAP^cre^p38α^−/−^ mice after intracerebral LPS injection

**Figure 8:**
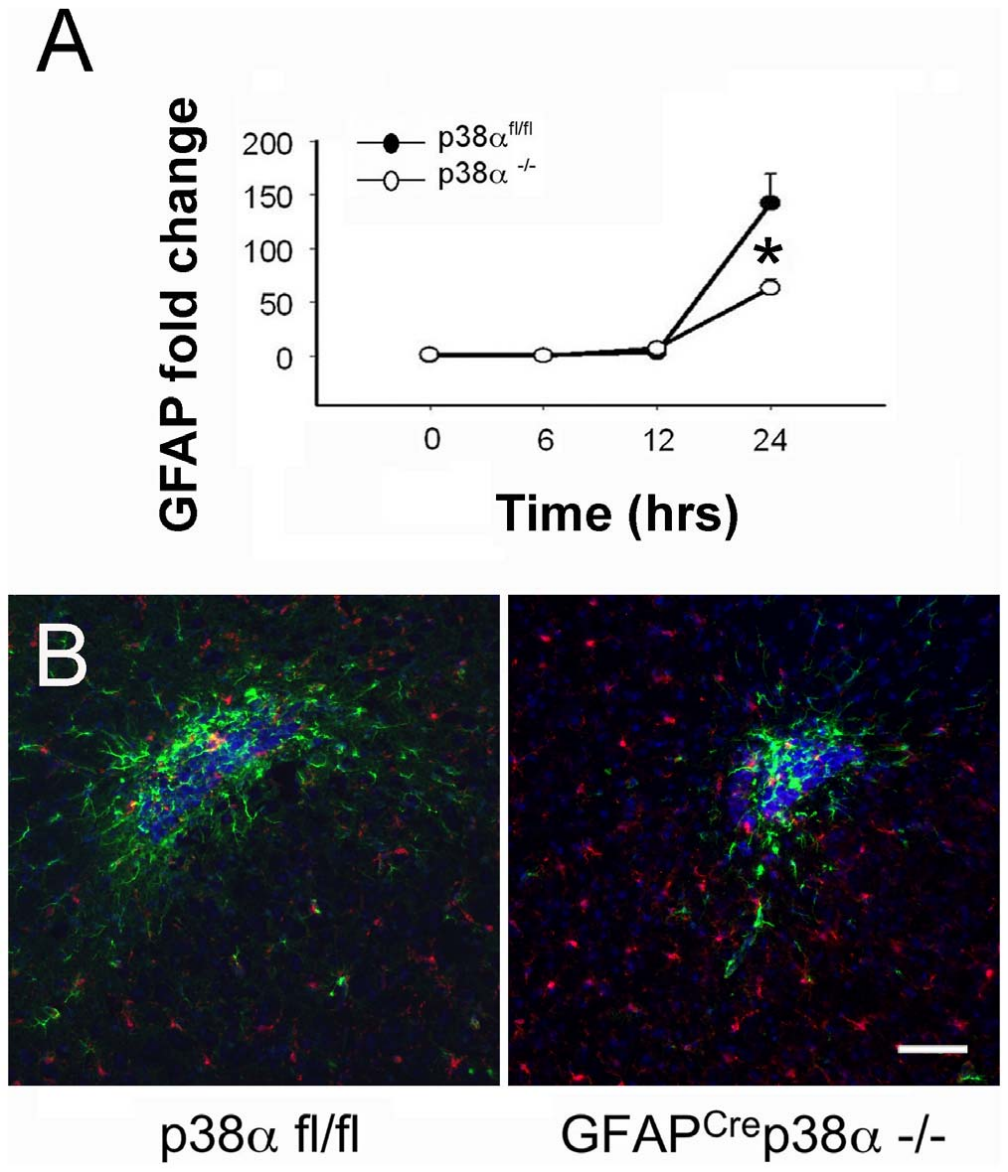
Detection of GFAP expression at the CNS site of injury in GFAP^Cre^ p38α^−/−^ mice after intracerebral LPS injection. (A) After intracerebral LPS injection, GFAP mRNA was significantly upregulated in both GFAP^cre^ p38α^−/−^ mice and p38α^fl/fl^ cohorts at 24 hours after LPS injection (p<0.05). But comparison between genotypes showed that this increase was significantly lower in GFAP^cre^ p38α^−/−^ mice compared to p38α^fl/fl^ cohorts (p < 0.05). (B) Strongly GFAP-immunoreactive astrocytes (green) characteristic of activation or “astrogliosis” were detected at the LPS injection site. Diffuse activation of Iba1 positive microglia/macrophage (red) was also observed in the proximity of the lesions. In GFAP^cre^p38α^−/−^ mice, astrocyte activation was markedly attenuated compared to p38α^fl/fl^ cohorts at 24 hours after intracerebral LPS injection. Iba1 positive cells did not show clustering at the site of injury, but rather distributed in the CNS parenchyma. Distribution of Iba1 positive microglia/macrophages was also found prominent in GFAP^cre^p38α^−/−^ compared to p38α^fl/fl^ mice (scale bar = 100 μm). Representative images from the two genotypes are shown. *p < 0.05; data represent mean ± SEM.

After an intracerebral LPS injection, we measured astrogliosis at the site of injection both indirectly by using astrocyte-specific mRNA responses and directly by immunohistochemical and histomorphological examination of astrocyte responses. At 12 and 24 hours after injections, we observed a significant upregulation of GFAP in both p38α^fl/fl^ and GFAP^cre^p38α^−/−^ mice (p < 0.05). However, the increases in GFAP mRNA in GFAP^cre^p38α^−/−^ mice were significantly lower compared to p38α^fl/fl^ cohorts at 24 hours after intracerebral LPS injection (p < 0.05; Fig. 8A). This was consistent with *in vitro* findings in which p38α^−/−^ astrocytes exhibited a relatively lower expression of inflammatory genes after LPS, IL-1β or IFNγ exposure. Supporting the qPCR data, immunohistopathology evidence also identified clear signs of reactive astrogliosis with associated microglia/macrophages in both GFAP^cre^ p38α^−/−^ and p38α^fl/fl^ mice 24 hours after LPS injection (Fig. 8B). However, the region of astrocyte activation and the entire dimensions of the lesion were markedly reduced in GFAP^cre^p38α^−/−^ mice. In p38α^fl/fl^ mice, we observed a dense population of GFAP-immunoreactive astrocytes with a characteristic morphology indicative of activation at the site of injection in the brain cortex. In GFAP^cre^p38α^−/−^ mice, the pathology was markedly less. These findings provide a second line of evidence indicating that astrocytes deficient in p38α are less responsive to inflammatory injury.

### Enhanced neutrophil infiltration in GFAP^cre^p38α^−/−^ mice after intracerebral LPS injection

**Figure 9:**
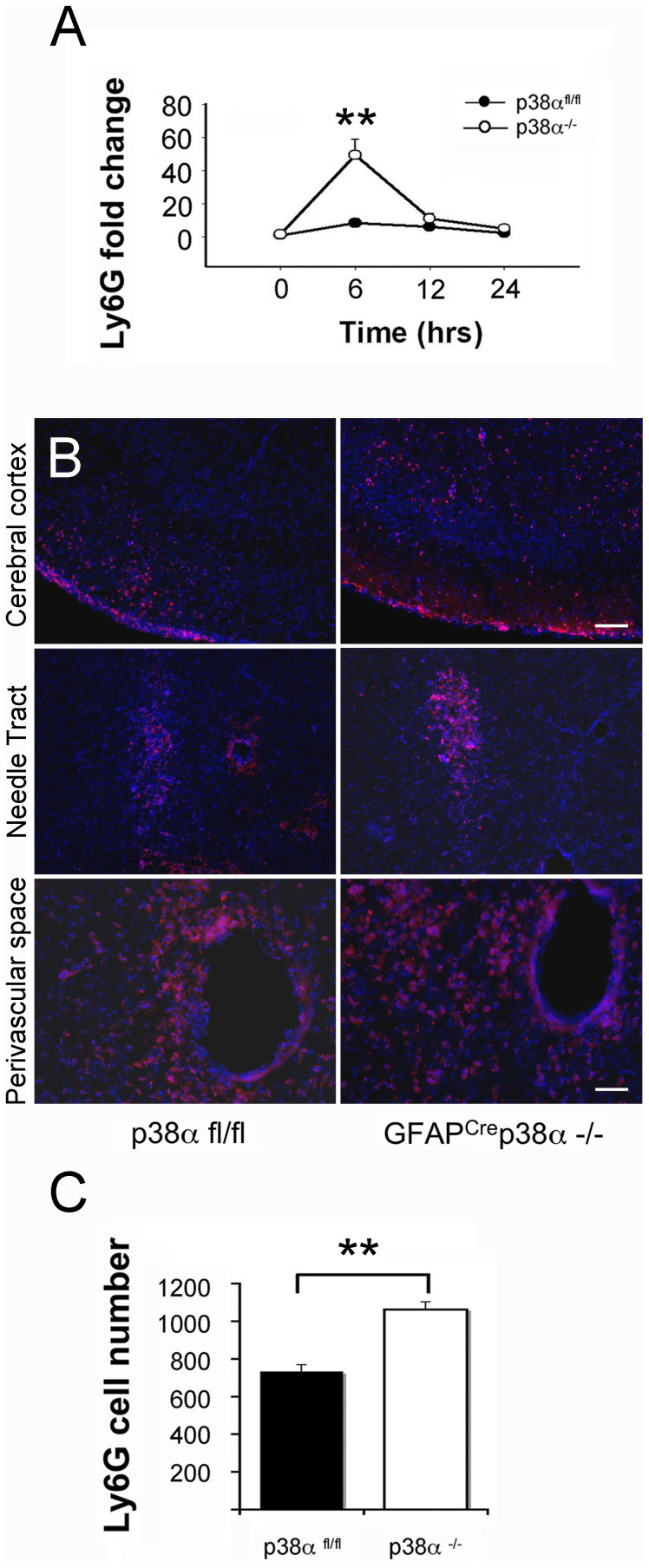
Neutrophil infiltration at the CNS site of injury in GFAP^Cre^ p38α^−/−^ mice after intracerebral LPS injection. (A) After intracerebral LPS injection, Ly6G mRNA showed significant upregulation in GFAP^cre^ p38α^−/−^ mice at 6 hours (p < 0.05); this upregulation was relatively mild and not significant in p38α^fl/fl^ cohort. A significantly higher Ly6G expression was occurred in GFAP^cre^p38α^−/−^ mice at 6 hours compared to p38α^fl/fl^ cohorts (p < 0.05). (B) Ly6G positive neutrophils were detected in the cortical region, LPS injection site (scale bar = 100 μm) and perivascular region (scale bar = 50 μm) in the brain parenchyma after intracerebral LPS injection. GFAP^cre^p38α^−/−^ mice showed marked increases in infiltrating neutrophils in all the three regions examined compared to p38α^fl/fl^ mice. (C) Quantification of Ly6G positive cells showed significantly increased numbers in the brains of GFAP^cre^ p38α^−/−^ mice compared to p38α^fl/fl^ mice. Representative images from the two genotypes are shown. **p < 0.001; data represent mean ± SEM.

To investigate the cellular source of upregulated chemokines and cytokines in GFAP^cre^p38α^−/−^ mice, we examined the presence and expression levels of Ly6G mRNA following intracerebral LPS injections. A significant upregulation of Ly6G mRNA was observed in GFAP^cre^p38α^−/−^ mice at 6 and 12 hours after intracerebral LPS injection (p < 0.05), while no observable upregulation of Ly6G was seen in p38α^fl/fl^ cohorts over time. In comparisons between the two genotypes, we detected a significantly higher expression of Ly6G mRNA in GFAP^cre^p38α^−/−^ mice at 6 hours after LPS injection compared to p38α^fl/fl^ cohorts (p < 0.05; Fig. 9A). After the initial increase at 6 hours, Ly6G mRNA expression returned to near-baseline levels at 12 hours post-LPS injection. This suggests an acute infiltration of neutrophils occurred at the CNS of GFAP^cre^p38α^−/−^ mice in response to an intracerebral LPS exposure. Supporting the qPCR data, immunocytochemistry evidence also identified a massive infiltration of neutrophils existing in both GFAP^cre^ p38α^−/−^ and p38α^fl/fl^ mice 24 hours after LPS injection (Fig. 9B). In particular, accumulation of Ly6G positive neutrophils surrounding the needle tract region was apparent in both p38α^fl/fl^ and GFAP^cre^ p38α^−/−^ mice. We observed a high density of Ly6G positive neutrophils at the perivascular space of the brain parenchyma in both p38α^fl/fl^ and GFAP^cre^ p38α^−/−^ mice. In contrast, the distribution of Ly6G positive neutrophils was dispersed at the cortical region of p38α^fl/fl^ and GFAP^cre^ p38α^−/−^ mice. Quantification of neutrophil infiltrates revealed a significantly increased number of Ly6G positive neutrophils present at the CNS of GFAP^cre^ p38α^−/−^ mice 24 hours post-LPS injection, compared to p38α^fl/fl^ cohorts (p < 0.01; Fig. 8C). Together these data provide strong evidence that there is significantly higher infiltration of neutrophils in the CNS of GFAP^cre^p38α^−/−^ mice after intracerebral LPS injection, compared to p38α^fl/fl^ cohorts.

### Increased microglia/macrophage presence in GFAP^cre^p38α^−/−^ mice after intracerebral LPS injection

**Figure 10:**
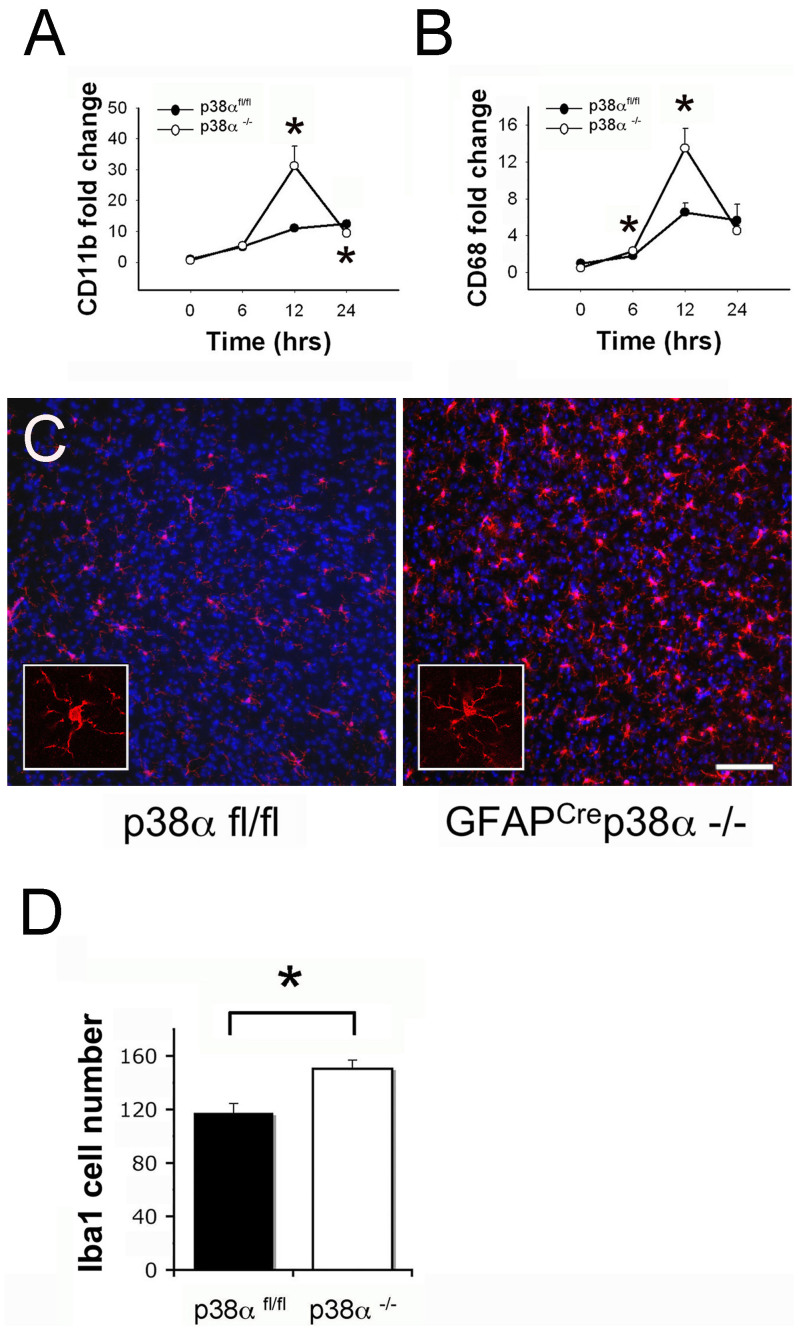
Microglia/macrophages at the CNS site of injury in GFAP^Cre^p38α^−/−^ mice after intracerebral LPS injection (A) After intracerebral LPS injection, CD11b mRNA showed significant upregulation in GFAP^cre^ p38α^−/−^ mice at 12 hours (p < 0.05); this upregulation was relatively moderate and not significant in p38α^fl/fl^ cohorts. Comparison between genotypes showed a significantly higher CD11b expression in GFAP^cre^p38α^−/−^ mice p38α^fl/fl^ cohorts at 12 hours (p < 0.05). (B) Similarly, CD68 mRNA showed significant upregulation in GFAP^cre^ p38α^−/−^ mice at 12 hours after LPS injection (p < 0.05); this upregulation was relatively moderate and not significant in p38α^fl/fl^ cohorts. Comparison between genotypes showed a significantly higher CD68 expression in GFAP^cre^p38α^−/−^ mice compared to p38α^fl/fl^ cohorts at 6 and 12 hours (p < 0.05). (C) Iba1 positive cells were distributed in the brain parenchyma and were not particularly clustered at the site of injury. A more prominent distribution of Iba1 positive microglia/macrophages were observed in GFAP^cre^ p38α^−/−^ mice compared to p38α^fl/fl^ cohorts at 24 hours after intracerebral LPS injection (scale bar = 100 μm). (D) Numerical quantification of Iba1 positive cells showed a significant increase in their numbers in GFAP^cre^ p38α^−/−^ mice compared to p38α^fl/fl^ control mice. *p < 0.05; data represent mean ± SEM.

Using CD11b and CD68 as specific markers for macrophages/microglia, we examined the inflammation site after intracerebral LPS injection in p38α^fl/fl^ and GFAP^cre^p38α^−/−^ mice. A significant upregulation of CD11b and CD68 mRNA was observed in GFAP^cre^p38α^−/−^ mice that increased with time and peaked at 12 hours after intracerebral LPS injection (p < 0.05); a relatively moderate but also significant upregulation of CD11b and CD68 was also seen in the p38α^fl/fl^ cohorts after LPS injection (p < 0.05). Interestingly, this upregulation of CD11b in GFAP^cre^ p38α^−/−^ mice was significantly higher than that observed in control p38α^fl/fl^ mice at 6 hours after LPS injection (p < 0.05; Fig. 10A). However, while the CD11b expression level for both genotypes dropped down at 24 hours after LPS injection, we observed significantly lower CD11b levels in GFAP^cre^ p38α^−/−^ mice at this time point, compared to p38α^fl/fl^ cohorts (p < 0.05; Fig. 10A). Corresponding with the expression pattern of CD11b, significantly higher expression levels of CD68 was observed in GFAP^cre^p38α^−/−^ mice at 6 and 12 hours after LPS injection, compared to p38α^fl/fl^ cohorts (p < 0.05; Fig. 10B). This observation corroborated that both primer sets accurately identified a specific cell type. Taken together, increased upregulation of CD11b and CD68 indicates either a more prominent CNS microglial proliferation and/or a greater number of macrophage infiltrations in the brain of GFAP^cre^p38α^−/−^ mice. To examine the specific tissue pathologies associated with intracerebral LPS injections, we performed immunohistochemistry for Iba1-positive macrophages/microglia in the CNS of both GFAP^cre^ p38α^−/−^ and p38α^fl/fl^ mice. Massive concentrations of macrophages/microglia were found present at the injection site in both p38α^fl/fl^ and GFAP^cre^ p38α^−/−^ mice (Fig. 10C). Quantification of Iba1 positive cells in this immunopathology revealed that significantly higher number of macrophages/microglia were present in the brain parenchyma of GFAP^cre^p38α^−/−^ mice compared to p38α^fl/fl^ cohorts 24 hours after intracerebral LPS injection (p < 0.05; Fig. 10D). Together, the differential effects between GFAP^cre^p38α^−/−^ and p38α^fl/fl^ mice highlight an important facet for p38α signaling in CNS inflammation.

In addition to neutrophil and macrophages/microglia, we investigated whether intracerebral LPS injection promoted T lymphocyte recruitment in the CNS parenchyma of GFAP^cre^ p38α^−/−^ and p38α^fl/fl^ mice. We examined the expression levels of CD4 and CD8 to identify T lymphocytes. We found no significant upregulation of CD4 or CD8 in both GFAP^cre^ p38α^−/−^ and p38α^fl/fl^ mice, compared to the non-injection p38α^fl/fl^ cohorts.

## Discussion

Signaling via the p38α pathway is essential for immune response in multiple cell types. As global p38α knockout mice show embryonic mortality^54^,^55^, the role of p38α in cell type specific responses *in vivo* has not been characterized. By generating conditional knockout mice, we sought to examine astrocyte-specific immune responses mediated by the p38α pathway. We revealed a critical role of p38α in the regulation of immunological responses and the production of specific cytokines and chemokines, and a rather complex role of astrocytes in immune regulation in the CNS. First, by isolating astrocyte-specific responses *in vitro*, we identified that p38α pathway critically regulates different cytokines: TNFα and IL-6, and chemokines: CCL2, CCL4, CXCL1, CXCL2 and CXCL10. By interpreting this information through what is known about the different cytokine and chemokine regulation, we believe that this regulation by the p38α pathway is by affecting multiple targets. It has been suggested that expression of CCL2, CXCL10 and IL-6 is via a NFκB-dependent mechanism^56^,^57^,^58^. In dendritic cells, it has been shown that p38 α activity is required at a very fundamental level for the phosphorylation and phosphoacetylation of histone H3 on a subset of cytokine and chemokine gene promoters in order to enhance accessibility to NFκB binding sites^59^,^60^. We found that p38α^−/−^ astrocytes are refractory to LPS treatment *in vitro* and there is a direct regulation of CCL2, CXCL10 and IL-6 by p38α signaling. The regulation of NFκB by p38α signaling in astrocytes would also induce secondary responses affecting multiple other effectors and adding to the complexity of the overall response. Other inflammatory mediators like CCL3, CCL4, CCL5, IL-1β and VCAM-1, also regulated at least in part by NFκB in different cell types^61^,^62^,^63^,^64^. CCL3, CCL5 and VCAM-1 induction was significantly lower in p38α^−/−^ astrocytes after IFNγ treatment, but induction was not different from p38α^fl/fl^ astrocytes after treatment with LPS or IL-1β. Similarly, CCL4 induction was significantly lower in p38α^−/−^ astrocytes after treatment with LPS or IL-1β, but induction was not different from p38α^fl/fl^ astrocytes after treatment with IFNγ. Although these specific effects provide direct clues to regulation of p38α signaling in astrocytes, deciphering the mechanisms underlying these observations is complicated by crosstalk between p38α and other immune-mediated signaling pathways that may result in different transcriptional responses.

Several reports have highlighted potential crosstalk between p38α, JNK and ERK1/2 pathways in many cell types^65^,^66^. p38α and JNK share an upstream kinase MKK4; in cancer cells, p38α deficiency has been suggested to cause MKK4 to shift its kinase activity toward JNK activation^67^. In astrocytes, we found that JNK activation is enhanced in p38α^−/−^ astrocytes, suggesting an aberrant effect in this pathway. JNK regulates c-Jun phosphorylation that is required for AP-1 transcriptional activation and gene expression^68^,^69^,^70^. It is known that overexpression of AP-1 induces ICAM-1and CCL2 up-regulation^71^, and that both JNK and c-Jun are required for CCL2 expression^72^. Findings from this study showed that up-regulation of ICAM-1 and CCL2 was not completely refractory in p38α^−/−^ astrocytes, suggesting a potential compensation due to enhanced JNK activation. Downstream effects of this crosstalk can be explained by the convergence of signaling leading to NFκB regulation.

When examining direct downstream targets of p38α upstream of NFκB, we detected a significantly lower expression of MK2 protein expression in p38α^−/−^ astrocytes. MK2 and MK3 are major protein kinases directly downstream of p38 MAPKs^35^, and studies have shown that p38α signaling is critical for cytokine-induced mRNA stabilization via MK2 and an AU-rich element (ARE) -targeted mechanism^51^. MK2 has been implicated in phosphorylation of ARE-binding proteins^73^, and a subsequent p38 MAPK-dependent stabilization of ARE is required for transcriptional activation of several inflammatory genes including IL-6, COX-2, TNFα and CXCL2^51^,^74^,^75^,^76^. Similar results have been reproduced in several other studies that demonstrate a direct link between MK2-mediated phosphorylation and the transcription of IL-6, TNFα and CXCL1^77^,^78^,^79^,^80^. In support of regulation mediated by MK2 downstream of p38α signaling, findings in this study showed significant down-regulation of TNFα, IL-6, CXCL1, CXCL2 and COX-2 in p38α^−/−^ astrocytes in response to different inflammatory stimuli. Taking another step in dissecting the role of p38α signaling in this response, we identified that LPS, IL-1β or IFNγ treatment all lead to a similar downregulation of CXCL1 in p38α^−/−^ astrocytes. This convergence between IFNγ receptor, IL-1 receptor and toll-like receptor triggered events suggests a possible role of a common adaptor element like MyD88; p38α and MK2 have been shown to be required for MyD88-mediated ARE-containing mRNA activation during chemokine expression^81^. Previous studies have demonstrated that MyD88 is an essential adaptor to transduce signaling cascades relayed from IFNγ receptor^81^, IL-1 receptor^82^, and the toll-like receptor 4 activity^83^. Findings in this study using p38α^−/−^ astrocytes recapitulated and validated this mechanism of regulation for CXCL1.

For further understanding of astrocyte regulation of immune function, we also examined the role of astrocyte p38α signaling in a more complex neuroinflammation context *in vivo*. Due to reduced expression of inflammatory genes observed in p38α^−/−^ astrocytes *in vitro*, we originally anticipated similar outcomes from the intracerebral LPS injection model in GFAP^cre^p38α^−/−^ mice. Unexpectedly, we found that the induction of chemokines: CCL2, CCL3, CCL4, CXCL2 and CXCL10, and cytokines: TNFα, IL-6 and IL-1β, and ICAM-1 were significantly higher in the CNS of GFAP^cre^p38α^−/−^ mice compared to p38α^fl/fl^ cohorts. Investigating the underlying reasons for this discrepancy revealed that GFAP^cre^p38α^−/−^ mice had significantly higher numbers of microglia and infiltrates like macrophages and neutrophils at the site of injury. Presence of these immune cells that were identified by specific markers Ly6G, CD11b and CD68 was correlated with the increased levels of cytokines and chemokines.

Many *in vivo* studies have suggested that neutrophils represent a significant source of chemokines such as CCL3, CCL4, CXCL1, CXCL8, and CXCL10^84^,^85^, which in turn chemoattract monocytes, dendritic cells, T lymphocytes and more neutrophils^23^,^86^. Therefore, neutrophils play a significant role in recruiting infiltrates at sites of CNS inflammation. Findings in this study indicated that GFAP^cre^p38α^−/−^ mice undergo acute and massive infiltration of neutrophils in response to CNS immune injury. This infiltration could be a cellular source that leads to significant up-regulation of CCL3, CCL4 and CXCL10 highlighting an important secondary effect due to the deficiency of p38α signaling in astrocytes. Perhaps as part of this neutrophil effect or an independent regulation, GFAP^cre^p38α^−/−^ mice develop a more prominent CNS microglia/macrophage response. Even though there are no markers to distinguish activation of resident microglia and the infiltrating macrophage populations, this observation reveals a clear difference of microglial/macrophage response in GFAP^cre^p38α^−/−^ mice in response to LPS. *In vitro* studies have shown that microglia/macrophages can produce TNFα and IL-1β in response to LPS stimulation^87^. Previous studies have also shown that microglia can produce CCL2, CCL3, CCL4, CCL5 and CXCL10^13^,^14^,^88^,^89^, and macrophages can produce CCL2, CXCL1, CXCL8^90^, in response to *in vitro* TNFα exposure. Therefore, increased microglial/macrophage activation observed in GFAP^cre^p38α^−/−^ mice could also contribute to the elevated levels of different cytokines and chemokines, adding to effects brought about by increased neutrophil infiltration.

We also observed a significant up-regulation of ICAM-1 in the CNS of GFAP^cre^p38α^−/−^ mice after LPS injections. It is well documented that interaction between ICAM-1 and β2 integrin CD11b/CD18 (MAC-1) is important for leukocyte transmigration at CNS inflammation sites^91^,^92^. This provides indirect evidence that there is a concurrent increase in macrophage infiltration in GFAP^cre^p38α^−/−^ CNS in addition to neutrophils, rather than a singular effect brought about by microglia. Altogether, our findings indicate that the deficiency of p38α attenuates an astrocytic response *in vitro* and results in an exacerbated CNS inflammatory response *in vivo*.

We directly examined astrocyte activation or “reactive astrogliosis” *in vivo* and showed an increase in GFAP expression both at the mRNA level and by immunohistopathology, suggesting that astrocytes in GFAP^cre^p38α^−/−^ mice were less responsive to inflammatory stress. This attenuation of astrocyte activity due to the deficiency of p38α interestingly led to robust neutrophil and microglial/macrophage recruitment to the site of CNS injury. In addition to providing significant insight into the specific role of p38α signaling in astrocyte activation during CNS inflammation, these findings show direct evidence that reactive astrocytes have a protective role restricting inflammation in the CNS.

We observed reduced inflammatory gene expression in p38α^−/−^ astrocytes *in vitro*, but higher expression of chemokines CCL2, CCL3, CCL4, CXCL2, CXCL10 and cytokines TNFα, IL-6, IL-1β as well as adhesion molecule ICAM-1 in the CNS of GFAP^cre^p38α^−/−^ mice after intracerebral LPS injection compared to p38α^fl/fl^ cohorts *in vivo*. Owing to the complexity of *in vivo* CNS inflammatory milieu, the source of upregulated proinflammatory cytokines, chemokines and adhesion molecules could be derived from multiple cellular components other than p38α^−/−^ astrocytes in the CNS of GFAP^cre^p38α^−/−^ mice. We found crucial evidence demonstrating a significantly increased expression of infiltrating cell markers such as Ly6G, CD11b and CD68 in the CNS of GFAP^cre^p38α^−/−^ mice, compared to p38α^fl/fl^ cohorts, indicating that *in vivo* p38α^−/−^ astrocytes with attenuated immune reactivity lost the capacity to maintain an intact astroglial barrier and thus result in an exacerbated permeability for leukocyte extravasation in the CNS parenchyma of GFAP^cre^p38α^−/−^ mice.

In accordance with attenuated astrogliosis of p38α^−/−^ astrocytes *in vivo*, a paralleled upregulation between infiltrating cell markers and subsets of specific chemokines and cytokines in GFAP^cre^p38α^−/−^ mice together confirmed that resident microglia, infiltrating neutrophils and macrophages are rather the major contributors of significantly upregulated proinflammatory cytokines and chemokines in the CNS of GFAP^cre^p38α^−/−^ mice. Our data demonstrates a significantly increased expression of neutrophil marker Ly6G paralleled with significant upregulation of CCL3, CCL4 and CXCL10 in the CNS parenchyma of GFAP^cre^p38α^−/−^ mice at identical time points after LPS injection, this suggests the cellular source of significantly upregulated CCL3, CCL4 and CXCL10 transcripts may be mainly derived from infiltrating neutrophils, rather than p38α^−/−^ astrocytes in the CNS of GFAP^cre^p38α^−/−^ mice. Following neutrophil invasion, macrophages and lymphocytes are the next infiltrating cell types recruited by the chemokines secreted from neutrophils or CNS resident cell types. A significantly increased amount of macrophage/microglia in the CNS parenchyma of GFAP^cre^p38α^−/−^ mice suggests either a more prominent CNS microglial proliferation or enhanced macrophage recruitment after intracerebral LPS injection. Thus, an elevated number of macrophages/microglia in the CNS of GFAP^cre^p38α^−/−^ mice may contribute to an increased upregulation of chemokines CCL2, CCL3, CCL4, CXCL10, and cytokine TNFα observed in GFAP^cre^p38α^−/−^ mice after LPS injection. Moreover, upregulation of ICAM-1 and CD11b in the CNS of GFAP^cre^p38α^−/−^ mice both peaked at 12 hours following intracerebral LPS injection. Interaction between ICAM-1 and β2 integrin CD11b/CD18 (MAC-1) is considered as an important component for leukocyte transmigration at CNS inflammation sites, suggesting that ICAM-1 upregulation has an indispensable role in an increased accumulation of CD11b^+^ macrophages/microglia in the CNS parenchyma of GFAP^cre^p38α^−/−^ mice. Particularly, a significant upregulation of CD11b and CD68 in the CNS of GFAP^cre^p38α^−/−^ mice may be partially derived from an additional recruitment of peripheral macrophages facilitated by an increased ICAM-1 expression.

Overall, we demonstrated consistency between *in vitro* and *in vivo* results in that astrocytes with p38α ablation are less reactive to inflammatory stimuli under both conditions. An attenuated immune reactivity of p38α^−/−^ astrocytes *in vivo* specifically indicates the relevance between diminished reactive astrogliosis and impaired astroglial barrier in the CNS of GFAP^cre^p38α^−/−^ mice. Meanwhile, in accordance with significantly increased Ly6G, CD68, and CD11b expression in the CNS of GFAP^cre^p38α^−/−^ mice, a contrasting result emerged between *in vitro* and *in vivo* inflammatory gene expression level together confirmed that microglia, infiltrating neutrophils and macrophages act as major contributors to those significantly upregulated cytokine and chemokine mRNAs in the CNS of GFAP^cre^p38α^−/−^ mice, rather than *in vivo* p38α^−/−^ astrocytes with less immune reactivity. Particularly, the phenomenon of significantly increased immune infiltrates in the brain parenchyma of GFAP^cre^p38α^−/−^ mice strongly suggests that the capacity of astrocytes in maintaining the CNS microvasculature barrier may be impaired due to p38α deficiency. On the other hand, this indicates that astrocyte p38α signaling may perform a more profound effect on regulating CNS microvasculature, rather than modulating immunological events under *in vivo* CNS inflammatory conditions.

We generated the conditional GFAP-specific p38α knockout mouse in order to study the definitive role of astrocyte p38α signaling under *in vivo* CNS inflammatory conditions. However, neurons derived from the neuroectoderm lineage underwent a GFAP-expression stage and have also been conditionally knocked-out of p38α during early neurogenesis^93^. Moreover, adult neurogenesis has been suggested to be mainly derived from GFAP-expressing progenitor cells^94^,^95^. Thus, there exists a p38α^−/−^ neuron population in addition to p38α^−/−^ astrocytes in the CNS of these GFAP^cre^p38α^−/−^ mice. During *in vitro* studies, the process of generating pure astrocyte cultures eliminated the p38α^−/−^ neurons and p38α^−/−^ neural progenitor cells through a sequential procedure of mechanical dislodgement, trypsinization and L-leucine methyl ester hydrochloride treatment. Therefore, a very minimal existence of p38α^−/−^ neurons does not interfere with the *in vitro* immune activity of p38α^−/−^ astrocytes in response to inflammatory stimulation. However, an inevitable existence of p38α^−/−^ neurons in the CNS of GFAP^cre^p38α^−/−^ mice might exhibit an additional effect on the *in vivo* CNS inflammatory response. Therefore, the existence of p38α deficient neurons and their response to LPS injection should also be considered as a potential factor contributing to the increased chemokine and cytokine upregulation in GFAP^cre^p38α^−/−^ mice in response to LPS-mediated inflammatory insults. Thus, neurons have the capacity to respond to an inflammatory stress via p38 MAPK signaling pathways and neurons with p38α ablation could be less vulnerable in response to inflammatory exposure *in vivo*. In our GFAP^cre^p38α^−/−^ mouse model, neurons with p38α deficiency being less responsive to LPS-induced inflammatory stress may reduce its production of cytotoxic signal molecules, leading to an attenuated astroglial reactivity under conditions of *in vivo* CNS inflammation. Taken together, an attenuated reactive astrogliosis *in vivo* due to either astrocyte or neuronal p38α deficiency may contribute to an impaired astroglial barrier at the CNS perivascular space, and consequently result in a massive leukocyte infiltration in the brain parenchyma of GFAP^cre^p38α^−/−^ mice following intracerebral LPS injection.

In conclusion, we identified a specific role of p38α in regulating specific subsets of inflammatory genes during an astrocytic immune response, and that astrocytes with p38α ablation are less responsive to inflammatory stress under both *in vivo* and *in vitro* conditions. Under CNS inflammatory conditions, a diminished reactivity of astrogliosis in GFAP^cre^p38α^−/−^ mice is suggested to impair the formation of astroglial barrier and abolish the effect on restricting leukocyte infiltration. Taken together, our findings reveal the significance of *in vivo* astrocyte p38α signaling in maintaining the barrier of CNS microcirculation, which is more crucial than modulating immunological events under CNS pathological conditions. Overall, this study not only highlights the importance of p38α signaling in astrocyte immune activation *in vitro*, but also provides mechanistic insight into understanding the cell-specific p38α signaling in the cellular components of neurovascular unit during CNS neuroinflammation. By investigating astrocyte function using the GFAP^cre^p38α^−/−^ mouse model, we resolve a long-existing controversy in the field regarding the outcome of astrocyte activation by providing direct evidence that reactive astrogliosis restricts the severity of inflammation in the injured CNS.

## Author Contributions

U.L., V.S., J.M.P. and O.V.C. designed and performed experiments; K.O. provided initial breeding pairs of p38α^fl/fl^ mice; U.L. and W.D. wrote the manuscript with input from all authors; W.D. directed the study.

## Supplementary Material

Supplementary Information
