# Supplementary material for: p38α (MAPK14) critically regulates the immunological response and the production of specific cytokines and chemokines in astrocytes
Source: Sci Rep. 2014 Dec 12;4:7405. doi: 10.1038/srep07405 (PMC4264013; doi:10.1038/srep07405)
Supplement: Supplementary Information [file srep07405-s1.pdf]

p38α (MAPK14) CRITICALLY REGULATES THE IMMUNOLOGICAL RESPONSE AND THE PRODUCTION OF SPECIFIC CYTOKINES AND CHEMOKINES IN ASTROCYTES

U-Ging Lo, Vimal Selvaraj, Jennifer M. Plane, Olga V. Chechneva, Kinya Otsu and Wenbin Deng

Supplementary Information

Table S1. Primer sets used for genotyping PCR

hGFAP-Cre	Forward	5'-ACT CCT TCA TAA AGC CCT-3'
	Reverse	5'-ATC ACT CGT TGC ATC GAC CG-3'
p38α floxed	Forward	5'-AGC CAG GGC TAT ACA GAG AAA AAC CCT GTG-3'
	Reverse	5-ATG AGA TGC AGT ACC CTT GGA GAC CAG AAG-3'
ROSA26-MT/MG	WT-Forward	5'-CTC TGC TGC CTC CTG GCT TCT-3'
	WT-Reverse	5'-CGA GGC GGA TCA CAA GCA ATA-3'
	Mutant-Reverse	5'-TCA ATG GGC GGG GGT CGT T-3'

Table S2. Inventoried assays used for quantitative PCR

Gene	Entrez Gene ID	Catalog number*
CCL2	NM_011333	QT00167832
CCL3	NM_011337	QT00248199
CXCL1	NM_008176	QT00115647
CXCL2	NM_009140	QT00113253
CXCL10	NM_021274	QT00093436
TNFα	NM_013693	QT00104006
IL-6	NM_031168	QT00098875
IL-1β	NM_008361	QT01048355
ICAM	NM_010493	QT00155078
VCAM	NM_011693	QT00128793
GFAP	NM_010277	QT00101143
CD68	NM_009853	QT00254051
Itgam	NM_008401	QT00156471
Ly6G	XM_909927	QT00529655

***** Catalog numbers are from Qiagen, Valencia, CA.

Primers designed for quantitative PCR

Gene	Entrez Gene ID	Sequence	
Rpl13a	NM_009438	Forward	5'-CAT TCT GGA GGA GAA ACG GAA GG-3'
		Reverse	5'-GCA GGC ATG AGG CAA ACA GTC-3'
CCL4	NM_013652	Forward	5'-TTC CTG CTG TTT CTC TTA CAC CT-3'
		Reverse	5'-CTG TCT GCC TCT TTT GGT CAG-3'
CCL5	NM_013653	Forward	5'-GCT GCT TTG CCT ACC TCT CC-3'
		Reverse	5'-TCG AGT GAC AAA CAC GAC TGC-3'
COX2	NM_011198	Forward	5'-ATG AGT GGT AGC CAG CAA AGC CTA-3'
		Reverse	5'-ACT GAG TAC CAG GCC AGC ACA AA-3'
